# Formulation of Personalized, Fortified Beverage Nanoemulsions for Space Exploration with Omega‑3 Polyunsaturated Fatty Acids

**DOI:** 10.1021/acsfoodscitech.5c01291

**Published:** 2026-04-08

**Authors:** Svenja Schmidt, Ian D. Fisk, Nicole Yang, Maria Saarela, Volker Hessel

**Affiliations:** † School of Chemical Engineering, 1066 Adelaide University, Adelaide 5005, Australia; ‡ ARC Centre of Excellence in Plants for Space, Adelaide University, Waite Campus, Urrbrae 5064, Australia; § Andy Thomas Centre for Space Resources, Adelaide University, Adelaide 5005, Australia; ∥ International Flavour Research Centre, University of Nottingham, Sutton Bonington Campus, Loughborough LE12 5RD, United Kingdom; ⊥ International Flavour Research Centre (Adelaide), Adelaide University, Waite Campus, Urrbrae 5064, Australia; # 95835 South Australian Research and Development Institute (SARDI), Adelaide 5001, Australia; ∇ School of Agriculture, Food, and Wine, Adelaide University, Waite Campus, Urrbrae 5064, Australia

**Keywords:** spontaneous emulsification, space foods, microfluidics, beverage emulsions, continuous processing, in-space manufacturing, fortified beverage formulation

## Abstract

Current food trends (i.e., personalization and fortification with bioactives) can reduce food monotony and health risks experienced by astronauts during space missions. Using beverage nanoemulsions (beverage emulsions: oil content ≤1 g/kg; nanoemulsions: diameter ≤200 nm for high bioavailability) as a formulation approach for fortified beverages, this study investigates (1) how product formulation influences the emulsification process (self-assembly of droplets via low-energy emulsification, i.e., spontaneous emulsification) compared to a model system, (2) how to establish beverage personalization, and (3) how to achieve future beverage production. An emulsion model system (oil phase: medium-chain triglyceride and polysorbate 80 at a surfactant-to-oil mass ratio of 1.0; aqueous phase: 5 mM phosphate buffer at pH = 7.4; 1 g/kg oil) with an average droplet size of 184 ± 12 nm (batch process) was successively extended with common beverage ingredients (acids, sugars, aroma) and fish oil rich in omega-3 fatty acids. While aroma compounds and acids have a subsidiary influence on emulsion characteristics, an increase in sucrose concentration increased droplet size and the incorporation of fish oil decreased droplet size. Six different beverage recipes (varying in aroma compound and sweetness level) with a dosage of 90 mg bioactive per 330 mL serving were developed for a recipe library for future beverage personalization. Translating the emulsification to a continuous microfluidic (channel diameter ≤1 mm) process led to a further decrease in droplet diameter (final emulsions: 80–120 nm). Thus, emulsification efficiency (droplet size in relation to surfactant use) can be increased both via appropriate ingredient selection and process mode.

## Graphical abstract

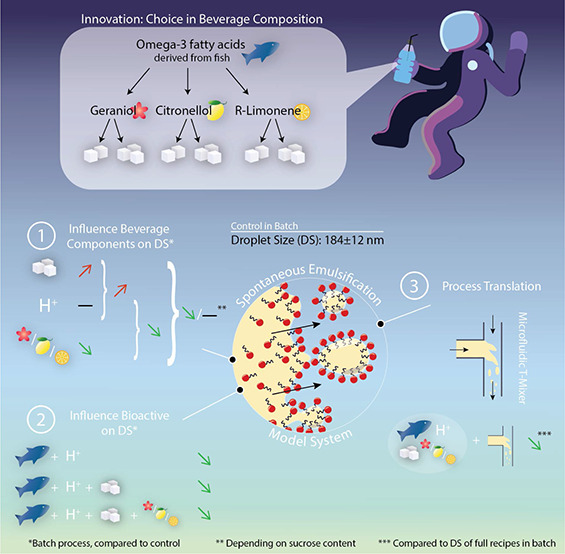

## 1 Introduction

Current space foods consist mainly of ultrastable food items, e.g., dehydrated and thermostabilized foods, which are combined in meals to meet energy and nutrition requirements.^1^ However, there have been various reports of astronauts not meeting the recommended caloric intake due to a lack of appetite experienced during space travel, i.e., space anorexia,^1,2^ suggesting that this approach is not suitable for the future of space travel, i.e., long-term (>6 months) space missions. In general, long-term space missions require novel approaches to space foods, since space shuttles lack the storage capacity to bring along a 3 years’ worth of food when traveling to Mars.^3^ Instead, researchers are working on space food systems that can produce or manufacture foods during space missions in the form of, e.g., in-space grown vegetables.^4^

Our research group follows an approach to provide astronauts with fortified, personalized beverages. Reviewing current trends in the food industry, we anticipate combining food personalization (i.e., novel beverage dispensers that offer a choice in flavor^5,6^ ) with food fortification^7,8^ to simultaneously address food monotony during space missions as well as space-specific health threats. Beverage personalization can be achieved by shifting the beverage production from prelaunch to in-space. Through appropriate process design, beverages can then be produced on demand, allowing one to address the astronauts’ personal preferences and/or nutritional needs during production. Specifically, we envision a building block approach, where different building blocks (beverage components, e.g., flavor and bioactives, see Figure 1A) are added to the beverage in different combinations, resulting in a variety of beverage compositions. We have termed this collection of beverage formulations a recipe library. Implemented in appropriate software, astronauts (or consumers on Earth) can access the library and choose the bioactives or micronutrients they require and the flavor they prefer.

**Figure 1:**
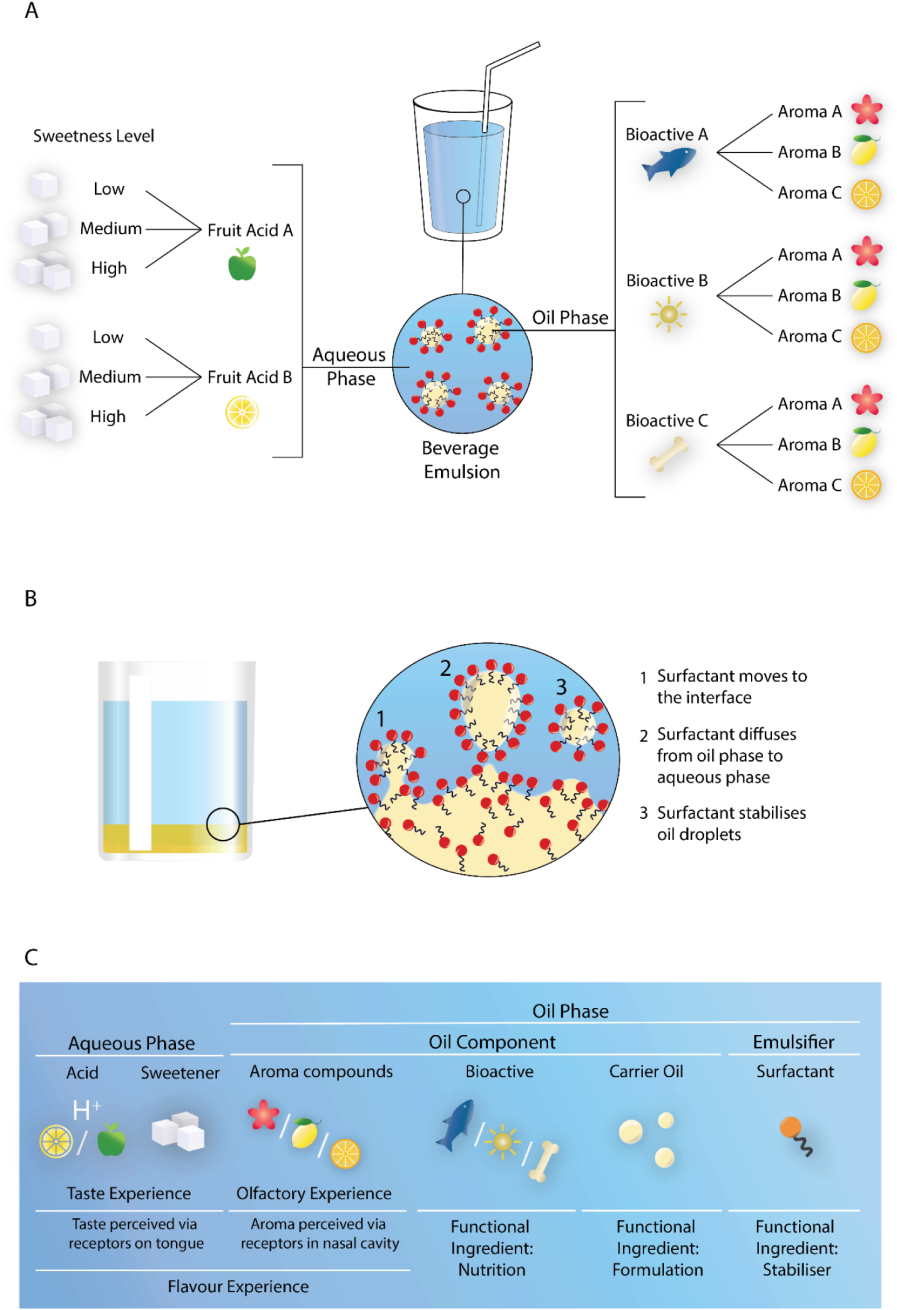
(A) Illustration of the building block approach, which serves as a design strategy for developing a recipe library of fortified, customizable beverages. (B) Illustration of spontaneous emulsification, a self-assembly emulsification method driven by the diffusion of the surfactant, which, in this case, is more hydrophilic than lipophilic. (C) Overview of the anticipated composition of a fortified beverage, with the aqueous phase containing both an acid (e.g., citric acid or malic acid) and a sweetener (e.g., sucrose), and the oil phase containing characteristic aroma compounds, bioactives as nutritional functional ingredients (derived from specific foods, e.g., seafood; counteracting space environmental characteristics, e.g., lack of sunshine; or targeting specific space health risks, e.g., reduction in bone density), a carrier oil as an aid for formulation design, and a surfactant to provide oil droplet stability.

Food fortification describes a process in which foods are enriched with nutrients or bioactives.^9^ Enriching the in-space-produced beverages with bioactives allows us to address personal nutrition deficiencies as well as general space health threats. Space-specific health threats are caused by conditions that define life in space, such as microgravity (e.g., bone and muscle loss), space radiation (e.g., oxidative stress, DNA damage), and isolation (e.g., stress).^10^ The approach of beverage fortification is intended not to replace standard foods but to complement daily nutrition. Due to the many positive effects of eating fresh and diverse foods for physical and mental health,^11^ NASA currently does not supplement nutrients or bioactives except for vitamin D, which is otherwise not available in a space diet.^12^ Yet, leading space agencies will consider bioactive supplementation once substantial evidence for a positive health effect, which cannot be achieved by normal nutrition, is available.^1^ One promising bioactive, omega-3 polyunsaturated fatty acid, might even be able to counteract two common space risks. First, when living in microgravity, the human muscles and skeleton do not need to provide resistance anymore against the gravitational force, resulting in muscle atrophy and increased rate of bone resorption.^13–15^ Second, when living outside the Earth’s protective atmosphere, astronauts are exposed to significantly higher levels of radiation, causing higher risks of cancer.^16^ Omega-3 polyunsaturated fatty acids, however, have been reported to potentially reduce bone resorption by increasing the rate of bone formation as well as decreasing the risk of some cancers.^17,18^ Supplementation or food fortification is likely to be required for omega-3 fatty acids, as they cannot be synthesized by the human body, while foods that are a typical source, for example sea foods, are not easily available during space missions, although current research is looking into adapting aquaponics as a food system for space.^19^ To maintain general health, astronauts are currently asked to consume 1.1 g/day (women) to 1.6 g/day (men) of omega-3 fatty acids in the form of standard foods, for example canned tuna, for missions up to 12 months.^12^ For achieving additional health benefits, i.e., prevention of bone mass loss and minimization of oxidative stress, the required amount of supplementation still needs to be identified.

For incorporating hydrophobic omega-3 polyunsaturated fatty acids into a beverage, beverage nanoemulsions are a promising formulation method, i.e., highly diluted oil-in-water emulsions (oil content ≤1 g/kg) with an average droplet diameter of less than 200 nm.^20^ Due to their higher surface-to-volume ratio, the droplet absorption into cells is considerably improved, resulting in higher bioavailability for encapsulated bioactives.^21,22^ An optimum in bioavailability is reached at an average droplet size of 50–100 nm.^23^ The nanostructure, however, has also caused concerns regarding the toxicity and biological fate of nanoemulsions.^23–25^ Droplets may be absorbed before reaching the intended site of action, causing adverse health effects. For food-grade nanoemulsions, however, oil droplets are most likely to be enzymatically digested in the upper gastrointestinal tract to monoglycerides and free fatty acids, i.e., common digestion products.^26^ Simultaneously, the extreme environments during ingestion (e.g., strong pH in the stomach) may also increase the average droplet size, preventing untimely absorption.^27^ In vitro and in vivo studies suggest that the main driver in nanoemulsion toxicity does not stem from their droplet size but rather from the increased absorption of potentially harmful composites, degradation products, or contaminants present in the nanoemulsion.^26^ So far, no adverse health effects have been identified for food-grade nanoemulsions; however, further contributions to the limited number of studies are advised to ensure consumer safety.^26^ For this study, we are focusing on beverage nanoemulsions as a formulation strategy due to their high bioavailability. Additionally, nanoemulsions are considered to be kinetically stable, providing higher protection against phase separation.^28^

To avoid using strong mechanical forces during nanoemulsification, which often lead to the formation of temperature gradients in the mixture,^29^ potentially damaging sensitive components, we are focusing on using low-energy emulsification, i.e., a solvent-free variant of spontaneous emulsification (see Figure 1B). In this case, the surfactant is soluble in both the oil and aqueous phases but has a higher hydrophilicity than lipophilicity. When the surfactant is initially dissolved in the oil phase, it diffuses into the aqueous phase upon contact, forming oil droplets in the process.^16,30^ Spontaneous emulsification typically requires synthetic surfactants with suitable hydrophilic–lipophilic balance (HLB);^30^ however, these surfactants do not provide nutritional value, and their use is typically regulated by national food agencies.^20^ For this study, the term oil phase will be used to describe the mixture of surfactant and the oil component, while oil component describes all hydrophobic, nonemulsifier components of the beverage emulsions, e.g., bioactive and aroma compounds (see Figure 1C).

Even though spontaneous emulsification is a self-assembly emulsification method, it is a slow process (range: hours) and is therefore typically combined with a convective mixing process. In this way, spontaneous emulsification is different from the self-assembly of thermodynamically stable microemulsions, which is a considerably fast process (range: minutes).^31^ Using a model system made of medium-chain triglycerides (oil), polysorbate 80 (surfactant), and phosphate buffer (aqueous phase), our lab has demonstrated that spontaneous emulsification can form stable nanoemulsions both when mixed via stirring in batch and via a continuous flow process.^32^ With the continuous process, surfactant use dropped by ∼50% to obtain nanoemulsions with an average droplet diameter of 100 nm. It was concluded that the microfluidic setup (T-mixer, internal diameter: 0.5 mm) used for the continuous process eased surfactant diffusion due to a higher relative interfacial area between the oil phase and the aqueous phase compared to the batch process. Microfluidics is a term in process engineering used to describe continuous and batch processes with channel diameters below 1 mm, achieving improved mass and heat transfer compared to conventional process design. Using this approach, surfactant use was within the acceptable limit recommended by both the FDA and the European Food Safety Authority for moderate consumption of beverage nanoemulsions (1 g polysorbate 80/kg; European Union limit: dietary foods for special medical purposes: 1000 mg polysorbates/kg;^33^ USA limit: in special dietary foods: not more than 360 mg polysorbate 80/day^34^).

The objective of this study is to elevate the formulation of the model emulsion to a fortified beverage nanoemulsion and investigate the implications on spontaneous emulsification. Typical beverage ingredients, as well as the bioactive, can ease or hinder the surfactant diffusion taking place during the self-assembly of droplets via spontaneous emulsification, which has been associated with smaller or larger droplets, respectively, in the final emulsion.^32^ To create a full-bodied flavor, both fortified and unfortified beverages typically contain a sweetener, an acid, and aroma compounds (see Figure 1C). The sweetener and the acid stimulate taste receptors on the tongue (taste experience), while volatile aroma compounds stimulate olfactory receptors in the ortho- and retronasal cavities (olfactory experience), which in combination result in the flavor experience. For space applications, carbonated drinks are avoided, as the lack of gravity prevents the gas bubbles from rising , creating an unpleasant mouthfeel.^35^

Sugars are most commonly used as sweeteners in beverages; for example, sucrose is widely used in Europe and Australia.^36^ Sucrose is a disaccharide consisting of glucose and fructose, which hydrolyzes into its components in the presence of acids.^37^ In fruit juices, different ratios of sucrose, glucose, and fructose are present.^38,39^ As carbohydrates, sugars could contribute to the daily caloric intake of astronauts and might help alleviate the effects of decreased appetite that astronauts have reported,^40^ although health risks associated with sugar consumption^41^ might necessitate the use of alternative sweeteners in the future, e.g., artificial sweeteners or rare sugars. Still, due to their widespread presence in common beverages, fructose, glucose, and sucrose at concentrations mirroring sugar contents in beverages and juices (100 g/L)^36,42^ were considered as sweeteners for this study.

Acids are added to beverages to provide tartness and tanginess,^43^ while additionally functioning as preservatives.^44^ Typically, organic fruit acids with a pH of 3 to 4 are used in beverage formulations because of their natural occurrence in fruits (e.g., citric acid: citrus fruits; malic acid: apples, pears, cherries; tartaric acid: grapes).^45^ For this study, we investigate the influence of citric acid and malic acid on emulsion characteristics due to their widespread use in fruit-flavored beverages.

Aroma compounds are volatile compounds with odor properties, which are perceived by olfactory receptor neurons located in the nasal cavity (orthonasal olfaction prior to consumption) and in the retronasal passage (retronasal olfaction during consumption). In most cases, an artificial aroma consists of a mixture of many different aroma compounds. For this study, we resolved to use single, hydrophobic compounds, which are known to provide a characteristic aroma: l-menthol (odor description: minty, cooling^46^ and odor threshold value in water (cooling): 0.95–1.85 mg/kg^47^), geraniol (odor description: rose-like^48^ and odor threshold value in water: 0.0075 mg/kg^49^), *R*-limonene (odor description: orange^50^ and odor threshold value in water: 0.034 mg/kg^51^), *S*-limonene (odor description: pine/turpentine^50^ and no odor threshold value in water reported), and β-citronellol (odor description: citrussy, fresh, floral^52^ and odor threshold value in water: 0.01 mg/kg^50^).

Carrier oils, in combination with an emulsifier, can help achieve a stable beverage formulation when adding hydrophobic ingredients. As medium-chain triglycerides have been reported to form the smallest droplet size among typical edible food oils during spontaneous emulsification,^30^ they are used in this study as the carrier oil. Medium-chain triglycerides are triglycerides composed of saturated medium-chain (6–12 carbon atoms) fatty acids, typically derived from fractionating coconut oil.^53,54^ Although medium-chain triglycerides are reported to increase satiety levels,^55^ it is unlikely that efficacious levels are reached when used as a carrier oil in beverage emulsions.^56^

To the authors’ best knowledge, the influence of these beverage-relevant constituents, as well as omega-3 fatty acids, on spontaneous emulsification has not yet been investigated. With these findings, we anticipate designing a preliminary recipe library for beverage personalization and implementing these recipes in a continuous microfluidic process for future in-space beverage production. The findings are also intended to support a future application of spontaneous emulsification in the terrestrial beverage industry.

## 2 Materials and Methods

### 2.1 Materials

Food-grade medium-chain triglycerides, obtained via fractionation of coconut oil by steam distillation^57^ (100% MCT Oil, Natures Aid Limited, Preston, UK), were purchased from a British health food shop (Holland & Barrett International Limited, Nuneaton, UK). In previous studies, pharmaceutical-grade medium-chain triglycerides, obtained via fractionation of coconut oil by controlled cooling,^58^ were used. A comparison between the two showed an increase in average droplet size (∼22%) in the batch process using food-grade medium-chain triglycerides , but no difference in average droplet size in the continuous microfluidic process (see Supporting Information). Fish oils rich in omega-3 polyunsaturated fatty acids were purchased in the form of fish oil soft capsules (Super Fish Oil Omega-3, Bioglan, Pharmacare (Europe) Limited, Weybridge, UK) from a British pharmacy (Boots UK Limited, Nottingham, UK). The fish oil was carefully extracted from the soft capsules using a syringe. According to the manufacturer, the fish oil contained 55% of docosahexaenoic acid (219.4 mg per 1 g of fish oil) and eicosapentaenoic acid (329.1 mg per 1 g of fish oil) combined, and a tocopherol-rich extract as an antioxidant. Polysorbate 80 and phosphate buffer (1.0 M, diluted to 5 mM with reverse osmosis water) were purchased from Sigma-Aldrich (Gillingham, UK, and Truganina, Australia). d-glucose anhydrous was purchased from Thermo Fisher Scientific UK (Loughborough, UK). d-(−)-fructose, sucrose for Sections 3.1.1 and 3.1.2, *S*-(−)-limonene, and l-menthol were purchased from Sigma-Aldrich (Gillingham, UK), with sucrose in the later part of the study being replaced by white sugar purchased from an Australian supermarket (Woolworths, Sydney, Australia). DL-malic acid (99+%) was purchased from Acros Organics (now Thermo Fisher Scientific UK, Loughborough, UK). Citric acid was purchased from SAFC Hitech Limited (Gillingham, UK) and Chem-Supply (Gillman, Australia). Geraniol (97%) and citronellol (95%) were purchased from Thermo Fisher Scientific (Heysham, UK). (*R*)-(+)-limonene was purchased from Sigma-Aldrich (Truganina, Australia).

### 2.2 Preparation of Oil Phase

The oil phase consisted of a mixture of oil components and a surfactant to enable spontaneous emulsification. The oil components consisted of medium-chain triglycerides as carrier oil, while a hydrophobic aroma compound and fish oil were added depending on the experiment (see Table 1). If an aroma compound (geraniol, *R*-limonene, or citronellol) was included in the oil phase, a quantity of 100 g/kg was added to the oil component, which relates to 100 ppm or 100 mg/kg aroma compound in the final emulsion (1 g/kg oil content). The concentration was chosen as this is above the reported aroma detection threshold for the aroma compounds in a water matrix, i.e., the concentration of the aroma compound is high enough to be sensed by the average consumer. Furthermore, concentrations are comparable to *R*-limonene naturally present in orange juice.^59^

**Table 1:** Overview of Composition of the Different Oil Phases Used in This Study^a^

			Oil phase with omega-3 fatty acids		
Components [g/kg]	Standard oil phase	Oil phase with aroma compound	50% in oil component	100% in oil component	Oil phase with omega-3 fatty acids and aroma compound
Oil component					
Medium-chain triglycerides	500	450	250	0	225
Aroma compound	0	50	0	0	50
Omega-3 fatty acids	0	0	250	500	225
Surfactant					
Polysorbate 80	500	500	500	500	500
Total	1000	1000	1000	1000	1000

a Quantities are given in g/kg oil phase

All oil components were mixed until thoroughly combined. Then, the surfactant, i.e., polysorbate 80, was added and mixed until thoroughly combined with the oil components. The surfactant concentration is described in the form of surfactant-to-oil ratio (SOR), which is based on the masses of the oil phase components (see eq 1). For all emulsions, the surfactant concentration was kept constant at an SOR of 1.0. This value was identified in a previous study as the lowest surfactant concentration required to form nanoemulsions in a continuous microfluidic process.^32^

1 $$\mathrm{SOR}=\frac{m_{\mathrm{Polysorbate}\;80}}{m_{\mathrm{Medium}‐\mathrm{chain}\mathrm{triglycerides}}+m_{\mathrm{Aroma}\mathrm{compound}}+m_{\mathrm{Fish}\mathrm{oil}}}$$

### 2.3 Preparation of Aqueous Phase

The aqueous phase consisted of a 5 mM phosphate buffer. Depending on the specific experiments, 25–100 g/L sugar (glucose, fructose, and sucrose) was dissolved in the aqueous phase. The sugar concentration was chosen to match the sugar concentration in both juices and sodas (20–120 g/L^42,60^ ). The pH was adjusted by adding either 1 M citric acid or 1 M malic acid to the aqueous phase.

### 2.4 Nanoemulsification via Batch Process

120 mg (±1 mg) portion of the oil phase was placed into a 100 mL beaker (see Figure 2A for experimental steps and setup). 60 g of the aqueous phase was added, and the mixture was stirred at 600 rpm for 20 min using a magnetic stirrer. Droplet size analysis was conducted immediately. Each emulsification experiment was conducted in triplicate.

**Figure 2:**
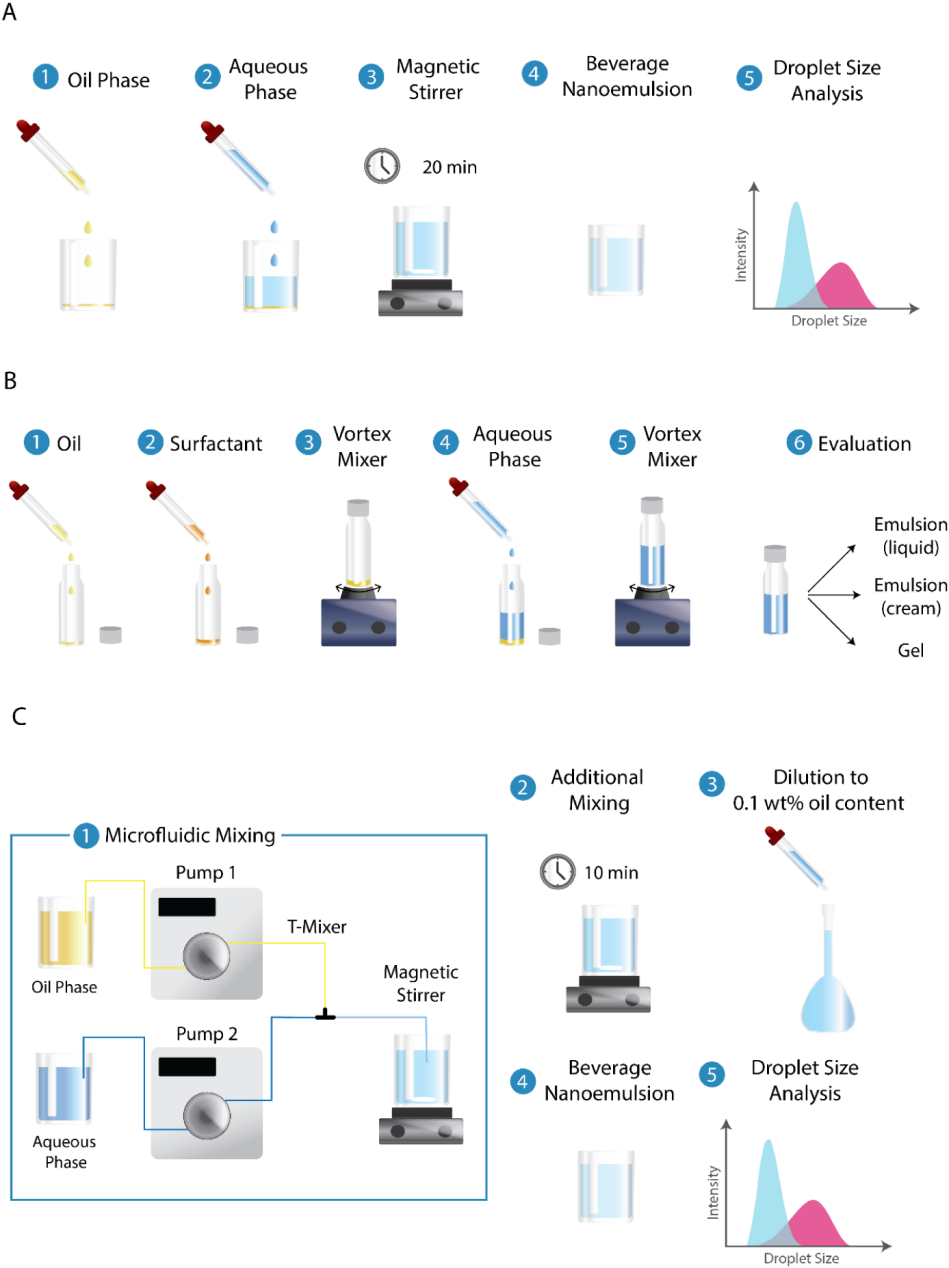
Overview of experimental procedures for different setups throughout this study. (A) Production of the fortified beverage emulsions using the batch process mode. (B) Preparation of an individual vial for the ternary phase studies. (C) Production of the fortified beverage emulsions using the continuous microfluidic process mode.

### 2.5 Preparation for Transfer in Continuous Microfluidic Process

#### 2.5.1 Characterization of 3-Phase System via Ternary Phase Diagram

In total, 6 different three-phase systems were studied (3 different oil components in combination with 2 different aqueous phases). The respective oil component contained a 1:1 ratio of medium-chain triglycerides and fish oil, as well as 100 g/kg of an aroma compound (*R*-limonene, citronellol, or geraniol). The aqueous phase (5 mM phosphate buffer) containing 50 and 100 g/L sucrose at pH 3 was prepared. For each 3-phase system (oil component, surfactant, and aqueous phase), 15 vials (2 mL HPLC glass vials) were prepared (see Figure 2B for the preparation steps of an individual vial). Each vial contained 1 g of the 3-phase system at different ratios (oil component: 10–50 wt %, surfactant: 10–50 wt %, and aqueous phase: 50–80 wt %; 10 wt % increments between vials; see Supporting Information for the composition of each vial and the composition of each 3-phase system). The oil component and the surfactant were first placed in the vial and thoroughly mixed using a vortex mixer. Then, the aqueous phase was added, and the mixture was again thoroughly mixed. The mixture in the vials was then categorized into (1) emulsion (liquid consistency), (2) emulsion (cream-like consistency), and (3) gel.

#### 2.5.2 Measurement of Density of the Oil Phase and Aqueous Phase

A calibrated vessel of defined volume was filled with the respective oil phase or aqueous phase (for the oil phase: 10 mL; for the aqueous phase: 20–25 mL). Its weight was determined before and after use on an analytical scale at room temperature (controlled at 21–23 °C). This step was repeated at least 10 times to achieve an accurate result. The average and standard deviation of the density of the respective phase were determined, and the density was expressed in g/mL.

#### 2.5.3 Determination of Volumetric Flow Rate for Oil Phase and Aqueous Phase

Assuming that pressure loss along the microfluidic mixing setup is negligible, the volumetric flow rate at the outlet, *V̇*_outlet_, was defined as the sum of the volumetric flow rate of the oil phase, *V̇*_oil phase_, and the volumetric flow rate of the aqueous phase, *V̇*_aq phase_, and was set to be 10 mL/min. 10 mL/min is a common upper limit for volumetric flow rates of microfluidic mixers and allows an easy comparison between different types of microfluidic mixers/setups. *V̇*_outlet_ allows the determination of the mass flow rate at the outlet, *ṁ*_outlet_ (*x*_oil phase, outlet_ = mass fraction of the oil phase at the outlet; ρ_oil phase_ = density of the oil phase; *x*_aq phase, outlet_ = mass fraction of the aqueous phase at the outlet; ρ_aq phase_ = density of the aqueous phase, assumed to be the density of water, i.e., 0.997 g/mL):

2 $${ṁ}_{\mathrm{outlet}}=\left(x_{\mathrm{oil}\mathrm{phase},\mathrm{outlet}}\;\rho_{\mathrm{oil}\mathrm{phase}}+x_{\mathrm{aq}\mathrm{phase},\mathrm{outlet}}\;\rho_{\mathrm{aq}\mathrm{phase}}\right){\mathrm{V̇}}_{\mathrm{outlet}}$$

As the oil phase consists of oil and surfactant, *x*_oil phase, outlet_ is the sum of the mass fraction of the oil in the outlet *x*_oil, outlet_ (100 g/kg or 10 wt % as per the experimental method, i.e., *x*_oil_ = 0.1), and the mass fraction of the surfactant at the outlet *x*_surfactant, outlet_. *x*_surfactant, outlet_ is the product of the SOR and *x*_oil, outlet_:

3 $$x_{\mathrm{oil}\mathrm{phase},\mathrm{outlet}}=x_{\mathrm{oil},\mathrm{outlet}}+x_{\mathrm{surfactant},\mathrm{outlet}}=x_{\mathrm{oil},\mathrm{outlet}}+\mathrm{SOR}\;x_{\mathrm{oil},\mathrm{outlet}}$$

*x*_oil phase, outlet_ allows the determination of the mass fraction of the aqueous phase at the outlet *x*_aq phase, outlet_:

4 $$x_{\mathrm{aq}\mathrm{phase},\mathrm{outlet}}=1-x_{\mathrm{oil}\mathrm{phase},\mathrm{outlet}}$$

*ṁ*_outlet_ and *x*_oil phase, outlet_ allow the determination of *V̇*_oilphase_, when defined via the volume fraction of the oil phase at the outlet, *y*_oil phase, outlet_:

5 $${\mathrm{V̇}}_{\mathrm{oil}\mathrm{phase}}=y_{\mathrm{oil}\mathrm{phase},\mathrm{outlet}}\;{\mathrm{V̇}}_{\mathrm{outlet}}=\frac{x_{\mathrm{oil}\mathrm{phase},\mathrm{outlet}}\;{ṁ}_{\mathrm{outlet}}}{\rho_{\mathrm{oil}\mathrm{phase}}}$$

Then, the volumetric flow rate of the aqueous phase at the outlet *V̇*_aq phase_ can be determined:

6 $${\mathrm{V̇}}_{\mathrm{aq}\mathrm{phase}}={\mathrm{V̇}}_{\mathrm{outlet}}-{\mathrm{V̇}}_{\mathrm{oil}\mathrm{phase}}$$

### 2.6 Nanoemulsification via a Continuous Microfluidic Process

For the continuous microfluidic process, a single-contact microfluidic mixer of simple geometry was used, i.e., a tee connection (or T-mixer) that brings both phases into contact. See Figure 2C for both the experimental setup and experimental procedure. The T-mixer had an internal diameter of 0.5 mm (Tee LP PEEK 1/4-28 1/16″ OD, 0.020″ ID, Upchurch Scientific, IDEX Health & Science, Lake Forest, USA), while all the tubing to and from the T-mixer had an internal diameter of 1 mm (1/16″ OD, 0.040″ ID). The short end of the T-mixer was connected to pump 1 (oil phase), while one of the long ends was connected to pump 2 (aqueous phase) (both pumps are Azura P 4.1S, 10 mL, Knauer, Berlin, Germany). The other long end functioned as the outlet, to which tubing of a length of ∼95 cm was connected. Pump 1 was flushed with ethanol prior to experimentation, as this reduces the risk of blockages in the tubing caused by the jellification of the oil phase at low water concentrations. The volumetric flow rate for pump 1 was set at 0.2 mL/min, and for pump 2 at 9.8 mL/min to reach an oil content of 10 g/kg in the final emulsion. To reach an oil content of 100 g/kg in the final emulsion, the volumetric flow rate was increased to 2 mL/min (pump 1) and 8 mL/min (pump 2). The pumps were run at the set volumetric flow rate for 7–10 min to reach equilibrium conditions, followed by a sample collection time of 5 min to collect a 50 mL emulsion sample in a 100 mL beaker. During sample collection, the mixture in the outlet beaker was stirred at 600 rpm. After sample collection, the mixture was stirred for an additional mixing time of 10 min at 600 rpm to allow thorough combination of both phases. If the formed emulsion contained 10 g/kg of oil, then 10 mL of the sample was diluted with the respective aqueous phase at a factor of 1:10 to reach an oil content of 1 g/kg. If the formed emulsion contained 100 g/kg, then 1 mL of the sample was diluted with the respective aqueous phase at a factor of 1:100 to reach an oil content of 1 g/kg.

### 2.7 Droplet Size Measurement

Droplet size measurement was conducted using a dynamic light scattering device (Zetasizer, Malvern Instruments Ltd., Malvern, UK). Due to the low oil content (1 g/kg), emulsion samples did not require dilution. The temperature during measurement was set to 25 °C. The average droplet size (droplet diameter) was recorded in the form of *z*-average, i.e., the intensity-weighted mean hydrodynamic droplet diameter. For simplicity, *z*-average will be referred to as droplet size in the Results and Discussion section. The dispersity index (formerly referred to as polydispersity index) was determined by the equipment’s software via a cumulative fit of the droplet size distribution to determine the degree of dispersity (dispersity index <0.1 for monodisperse droplets). Each emulsion replicate was measured in triplicate.

### 2.8 Statistical Analysis

The *z*-average and the dispersity index of each data point (i.e., emulsions prepared under the same parameters) were tested for outliers using a Dixon test (two-sided, significance level of 5%). Values with a *z*-score lower than −1.96 or higher than 1.96 were removed. Statistical significance between the emulsification parameters was identified by applying analysis of variance (ANOVA; 95% confidence interval) in combination with a Tukey pairwise comparison. All statistical tests were performed using XLSTAT (Life Sciences version 2023.1.6, XLSTAT statistical and data analysis solution; Lumivero, Denver, USA). The average and standard deviation were determined for each data point.

## 3 Results and Discussion

### 3.1 Influence of Emulsion Formulation on the Spontaneous Emulsification

#### 3.1.1 Influence of Individual, Fortified Beverage Ingredients on the Spontaneous Emulsification

##### 3.1.1.1 Sweetener=Sugars: Fructose, Glucose, and Sucrose

Without the addition of sugar in the aqueous phase, the model emulsion system (medium-chain triglycerides as oil, polysorbate 80 as surfactant, and 5 mM phosphate buffer as aqueous phase) formed via spontaneous emulsification in the batch process led to an opaque oil-in-water mixture with an average droplet size of 184 ± 12 nm. It was identified as a nanoemulsion, as both the dispersity index (0.33 ± 0.08) and the width of the particle size distribution (see Figure 2 in the Supporting Information) did not align with microemulsions (dispersity index <0.1 at narrow, monodisperse particle size distribution), while the average particle size exceeded typical values for micelles (2–50 nm). Even though the average particle size for the obtained nanoemulsion was below the wavelength of visible light, a fraction of the droplets were within the range of the wavelength of visible light, causing light scattering and the associated opaque appearance.

Increasing fructose, glucose, or sucrose concentration slightly (25 g/L) did not significantly impact the emulsions’ droplet size (fructose, glucose; ANOVA, 95% confidence interval) or led to a slight decrease in droplet size (sucrose: −9%). Increasing fructose, glucose, or sucrose concentrations to moderate to high concentrations (50–100 g/L) increased droplet size in general to values above 200 nm, therefore being outside the targeted droplet size for nanoemulsions. A linear relationship between sugar concentration and droplet size was not observed; rather, the droplet size increased stepwise. For fructose as a sweetener, the highest droplet size of 219 nm was reached at a concentration of 50 g/L (an increase of 18.5% compared to the control; see Figure 3A). For glucose and sucrose, the highest droplet sizes of 227 ± 31 nm and 228 ± 33 nm, respectively, were reached at a concentration of 100 g/L (an increase of 24% and 23%, respectively, compared to the control; see Figure 3C and E). Simultaneously, the dispersity index ranged from 0.26 to 0.35 for all emulsions, with no (fructose) or little impact (glucose, sucrose) from sugar concentration. The values themselves indicate a low-to-medium degree of polydispersity of the emulsions (see Figure 3B–F), while the approximately consistent dispersity index indicates there is no abrupt increase in the range of droplet sizes present in the emulsions. Rather, particle size distribution either shifted in its entirety to higher droplet sizes or, alternatively, stayed within the same boundaries but its area shifted toward higher values, both resulting in larger droplet sizes. Either way, the addition of sugars above 25 g/L seems to hinder the self-assembly of droplets driven by surfactant diffusion from the oil phase to the aqueous phase during spontaneous emulsification, leading to overall larger droplets. It is likely that the hindered surfactant diffusion is linked to the increased density and viscosity of the aqueous phase from addition of sugars,^61,62^ which slows down molecular movement in general.

**Figure 3:**
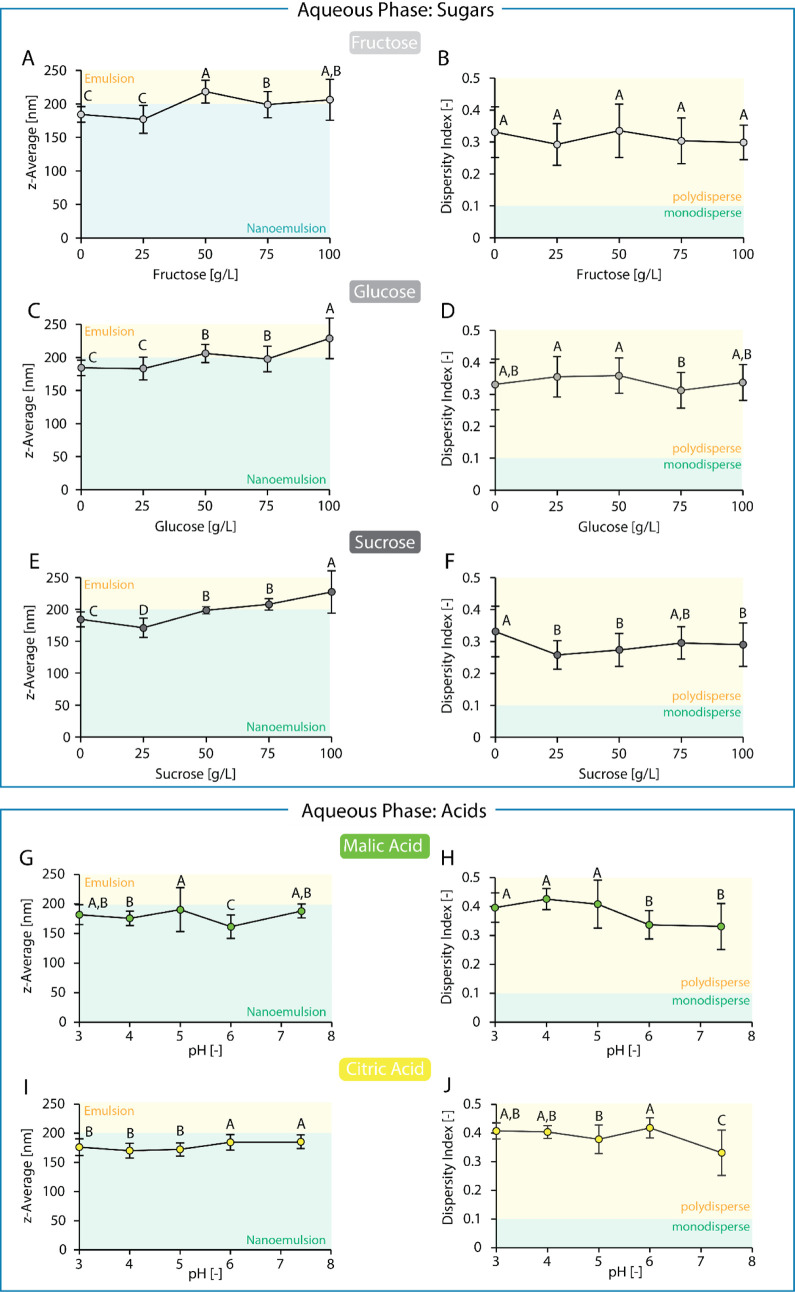
Influence of increasing the concentration of fructose, glucose, and sucrose in the aqueous phase on average droplet size in the form of *z*-average (A, C, E) and on dispersity index (B, D, F). Influence of decreasing pH of the aqueous phase (adjusted with citric acid) on average droplet size in the form of *z*-average (G) and on dispersity index (H). Influence of decreasing pH of the aqueous phase (adjusted with malic acid) on average droplet size in the form of *z*-average (I) and on dispersity index (J). All emulsions contained medium-chain triglycerides and polysorbate 80 as the oil phase (SOR = 1.0) and 5 mM phosphate buffer as the aqueous phase. All emulsions were prepared via the batch process at 1 g/kg oil content. Statistical significance was identified via ANOVA statistical testing (95% confidence interval) in combination with Tukey pairwise comparison. Statistically significant results are indicated by different letters, with “A” assigned to the overall highest value(s), “B” to the subsequent smaller value(s), and so forth. Reported are the average values with standard deviation.

For the recipe library, sugar levels of 50 and 100 g/L were considered to offer two sweetness levels of different intensity (medium–high). Sucrose, as sweetener, was chosen because of its widespread use in sodas and fruit drinks in the beverage industry.

##### 3.1.1.2 Acids: Citric Acid and Malic Acid

Upon the low addition of malic acid (pH = 6) to the model emulsion system, the droplet size first decreases to 162 ± 12 nm (a decrease of 12%). However, further addition of malic acid (pH = 3–5) increased droplet size so that the droplet sizes were not significantly different (ANOVA, 95% confidence interval) from the droplet size at pH = 7.4 (184 ± 12 nm; see Figure 3G). In contrast, a medium addition of citric acid was required to reduce droplet size slightly (a decrease of 7% at pH = 5), but further addition of citric acid did not influence droplet size (173 ± 13 nm at pH = 3–5; see Figure 3I). In both cases, the addition of acid led to an increase in the dispersity index (on average ∼20% in the pH range of 3–5 for malic acid and 3–6 for citric acid; see Figure 3H and J). The higher dispersity index upon addition of fruit acids indicates that the droplet size distribution widens, with more droplets present at very small or very large diameters. Thus, while acidic environments in some cases (citric acid) seem to support the self-assembly of droplets during spontaneous emulsification, as indicated by smaller average particle sizes, the droplet formation also becomes in general more irregular, causing the formation of particles with widely ranged diameters.

For recipe development, a pH value of 3 was considered because it best represents the variety of beverage products on the market. On average, 3.7 g/L of citric acid was required to achieve a pH value of 3, which is within the range of citric acid concentrations found in commercial soft drinks.^63^ Furthermore, the use of citric acid complements the aroma compounds considered in the next section, which are partly derived from citrus fruits.

##### 3.1.1.3 Aroma Compounds: l-Menthol, Geraniol, *R*-Limonene, *S*-Limonene, and Citronellol

Upon addition of 100 ppm (=100 mg/kg) aroma compound to the model emulsions’ compositions, the droplet size decreased in general, with all obtained emulsions being within the nanoemulsion range. Depending on the respective aroma compound, droplet size decreased by 15–27% (135–156 nm), even though the aroma compound made up only 5% of the oil phase. The addition of citronellol achieved the lowest droplet size, followed by *S*-limonene, *R*-limonene, geraniol, and l-menthol (see Figure 4A). The addition of the aroma compound also decreased the dispersity index by 26% (0.25), i.e., it led to a narrowing of the particle size distribution of low-polydisperse emulsions. Yet, the dispersity index did not show a dependency on the aroma compound used (see Figure 4B).

**Figure 4:**
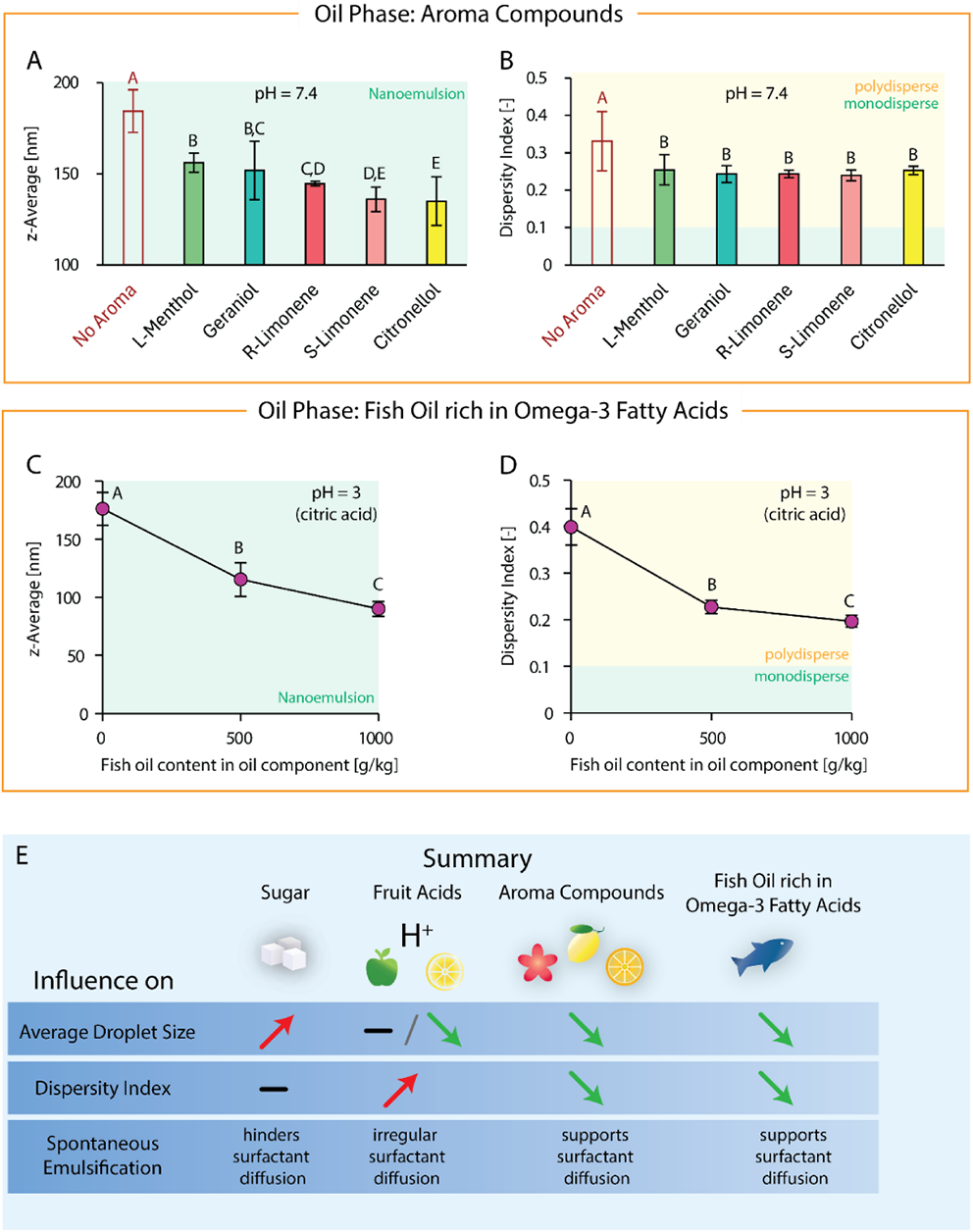
Top: Comparison of the influence of different aroma compounds (l-menthol, geraniol, *R*-limonene, *S*-limonene, and citronellol) on average droplet size in the form of *z*-average (A) and dispersity index (B) at pH = 7.4. Aroma compounds were added at 100 g/kg to the oil component of the oil phase (other constituents: medium-chain triglycerides, polysorbate 80, and SOR = 1.0), resulting in a concentration of 100 ppm in the final emulsion. Influence of fish oil content in the oil component of the oil phase (other constituents: medium-chain triglycerides, polysorbate 80, SOR = 1.0) on average droplet size in the form of *z*-average (C) and dispersity index (D) at pH = 3, adjusted with citric acid. All emulsions contained 5 mM phosphate buffer as the aqueous phase and were prepared via the batch process at 1 g/kg oil content. Statistical significance was identified via ANOVA statistical testing (95% confidence interval) in combination with Tukey pairwise comparison. Statistically significant results are indicated by different letters, with “A” assigned to the overall highest value(s), “B” to the subsequent smaller value(s), and so forth. Reported are the average values with standard deviation. (E) Summary of the influence of different beverage-specific substrates on emulsion characteristics and spontaneous emulsification.

Volatile aroma compounds typically have lower viscosities and densities^64^ compared to nonvolatile organic compounds. When incorporated into the oil phase, they are likely to decrease both the density and viscosity of the oil phase. Lower densities and viscosities are likely to ease droplet diffusion from the oil phase to the aqueous phase during spontaneous emulsification, thereby facilatating the self-assembly of droplets and resulting in smaller droplet sizes and a narrow particle size distribution.

##### 3.1.1.4 Bioactive: Fish Oil Rich in Omega-3 Polyunsaturated Fatty Acids

Incorporation of fish oil had the strongest relative impact on the average particle size and dispersity index of all beverage ingredients considered for this study. At 50% fish oil concentration in the oil component, the droplet size dropped by 35% to 114 ± 14 nm, even though fish oil only made up 25% of the oil phase. At 100% fish oil concentration in the oil component (50% fish oil in the oil phase), droplet size dropped by 49% to 90 ± 6 nm (see Figure 4C). Simultaneously, the dispersity index decreased by 43% (50% fish oil in the oil component) to 48% (100% fish oil in the oil component) to ∼0.2 (see Figure 4D), i.e., polydisperse emulsions of considerably narrow particle size distribution.

The addition of fish oil seems to ease surfactant diffusion during spontaneous emulsification considerably, which is associated with the considerably smaller average droplet size. Fish oil has, in contrast to the aroma compounds, approximately the same density and higher viscosity as medium-chain triglycerides (fish oil: density: 0.93 g/mL at 20 °C, viscosity: 50 mPas at 20 °C;^30,65^ medium-chain triglycerides: density: 0.95 g/mL at RT, viscosity: 32 mPas at 20 °C^30,32^ ), thereby making it unlikely to be a factor in enhancing diffusion. Fish oil has a slightly lower interfacial tension compared to medium-chain triglycerides (fish oil: 24.4 mN/m, medium-chain triglycerides: 28.2 mN/m; against water^30^), which, in general, can support mass transport from oil to water. On a molecular level, omega-3 fatty acids are present as triglycerides in fish oil.^66^ Due to their longer chain length (16–24 carbon atoms) compared to medium-chain fatty acids (6–12 carbon atoms), omega-3 triglycerides have a higher molecular weight than medium-chain triglycerides, which typically hinders diffusion. Instead, the double bonds present in the polyunsaturated, aliphatic tails of omega-3 triglycerides may be the main reason for eased diffusion compared with the saturated medium-chain triglycerides. The double bonds provide an increased flexibility to the omega-3 triglyceride molecules compared to the more rigid structure of saturated medium-chain triglyceride molecules,^67^ thereby likely to support diffusion during spontaneous emulsification and leading to overall smaller droplet sizes and dispersity indices. For a summary of the influence of individual beverage ingredients on spontaneous emulsification, see Figure 4E.

For beverage fortification, the maximum potential supplementation is ∼515 mg of docosahexaenoic and eicosapentaenoic acid combined per liter of fortified beverage (oil component is 100% fish oil, added at 1 g/kg in the final emulsion). Replacing medium-chain triglycerides only partly with fish oil (oil component is 50% fish oil and 50% medium-chain triglycerides, added in total at 1 g/kg in the final emulsion) leads to ∼258 mg/L of omega-3 fatty acid supplementation. Assuming a standard drink size of 330 mL, fortified beverage emulsions can cover 40–70% (95–170 mg) of the recommended daily amount of omega-3 fatty acids. For recipe development, 50% of fish oil in the oil component was considered, resulting in a supplementation of 258 mg/L of omega-3 fatty acids. This concentration provided the greatest decrease in particle size relative to its proportion in the oil phase. Additionally, the combination of medium-chain triglycerides and omega-3 fatty acids has been demonstrated to provide better uptake of omega-3 fatty acids into cell membranes.^68^ In the future, higher omega-3 fatty acid supplementation can be achieved by using fish oil with a higher omega-3 fatty acid content.

#### 3.1.2 Influence of Fortified Beverage Ingredients in Combination on the Spontaneous Emulsification

##### 3.1.2.1 Aqueous Phase: Combination of Sucrose and Acid

When both beverage ingredients, sucrose (100 g/L) and fruit acid (pH = 3), are present in the aqueous phase of the beverage emulsion, both of their previously observed individual effects on emulsion characteristics remain: sucrose increases droplet size (hindering surfactant diffusion), while acid increases the dispersity index (irregular surfactant diffusion increases the range of droplet sizes). For malic acid, the combination of both ingredients even led to a stronger increase in both characteristics than when sucrose was incorporated individually. Droplet size increased by 51% to 260 ± 39 nm (compared to 23% for sucrose incorporation; see Figure 5A) and the dispersity index increased by 37% to 0.46 ± 0.04 (compared to a decrease of 12% for sucrose incorporation; see Figure 5B). In contrast, citric acid in combination with sucrose does not exceed the effects observed individually, as the values of the droplet size and the dispersity index are not statistically significant compared to the control (ANOVA, 95% confidence interval; see Figure 5C and D). In both cases, the obtained emulsions are outside the targeted droplet size range, i.e., nanoemulsions. Additional measures (formulation, production) need to be taken to achieve a decrease in droplet size to reach the anticipated size range. For recipe development, citric acid was favored due to the absence of synergistic effects and its compatibility with many aroma compounds.

**Figure 5:**
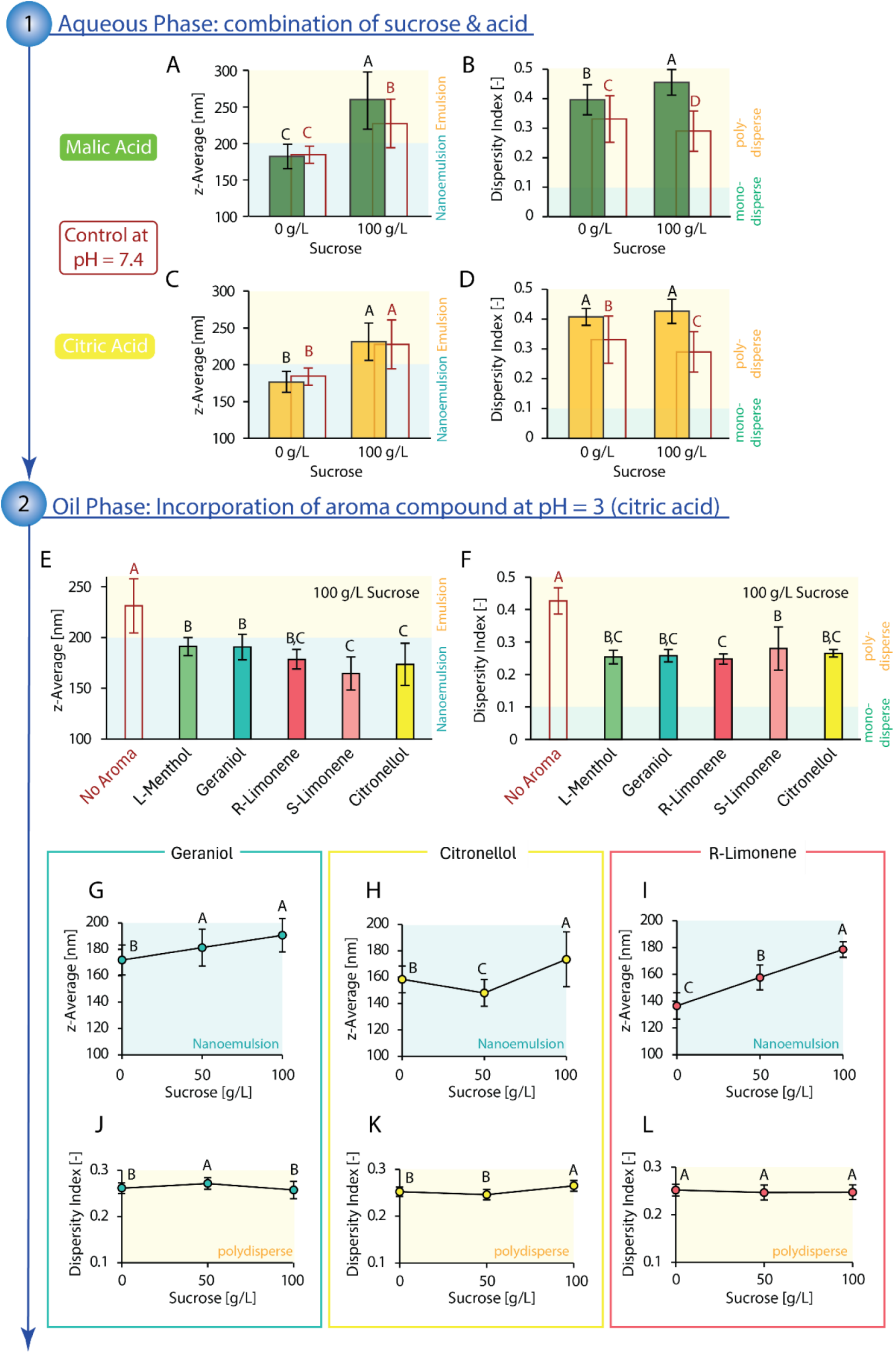
Top: Influence of increasing sucrose concentration and acid (red outline: control at pH = 7.4, green box: malic acid at pH = 3, yellow box: citric acid at pH = 3) on average droplet size in the form of *z*-average (malic acid: A; citric acid: C) and on dispersity index (malic acid: B; citric acid: D). The oil phase consisted of medium-chain triglycerides and polysorbate 80 at SOR = 1.0. Middle: Comparison of the influence of different aroma compounds (l-menthol, geraniol, *R*-limonene, *S*-limonene, and citronellol) on average droplet size in the form of *z*-average (E) and dispersity index (F) at pH = 3 (citric acid) and 100 g/L sucrose in the aqueous phase. Bottom: Influence of sucrose concentration on emulsion characteristics for emulsions (pH = 3, citric acid) containing geraniol (*z*-average: G, dispersity index: J), citronellol (*z*-average: H, dispersity index: K), and *R*-limonene (*z*-average: I, dispersity index: L). Aroma compounds were added at 100 g/kg to the oil component of the oil phase (other constituents: medium-chain triglycerides, polysorbate 80, SOR = 1.0), resulting in a concentration of 100 ppm in the final emulsion. All emulsions were prepared via the batch process at 1 g/kg oil content. Statistical significance was identified via ANOVA statistical testing (95% confidence interval) in combination with Tukey pairwise comparison. Statistically significant results are indicated by different letters, with “A” assigned to the overall highest value(s), “B” to the subsequent smaller value(s), and so forth. Reported are the average values with standard deviation.

##### 3.1.2.2 Oil Phase: Incorporation of Aroma Compound

The incorporation of aroma compounds (l-menthol, geraniol, *R*-limonene, *S*-limonene, citronellol) into the oil phase (100 g/kg in the oil component), in addition to sucrose (100 g/L) and citric acid (pH = 3) in the aqueous phase, eased surfactant diffusion in alignment with previous observations without sucrose and citric acid. Depending on the individual aroma compound, droplet size decreased by 17–29% from 231 ± 27 nm (no aroma) to 165–191 nm (see Figure 5E), approximately the same relative decrease as in the absence of sucrose and citric acid (15–27%). The observed decrease in droplet size was even sufficient to reach the anticipated nanorange emulsions. While not completely negating the droplet size-increasing effect of sucrose (see Figure 5G,H,I, where the addition of sucrose generally increased droplet size in the presence of aroma compounds), some aroma compounds were able to mitigate the relative increase in droplet size. Especially for geraniol and citronellol, the droplet size increased only by 10% instead of 23% (no aroma compound). For citronellol, the droplet size decreased even by 6% at 50 g/L sucrose. For *R*-limonene, however, droplet size increased by 31% compared to 23% (no aroma compound) at a sucrose content of 100 g/L, surpassing the effect of sucrose alone.

The dispersity index also decreased upon the addition of aroma compounds by 34–43%, from 0.43 (no aroma) to 0.25–0.28 (see Figure 5F), exceeding the previously observed effect of 26% in the absence of sucrose and citric acid. The combination of, particularly, the aroma compound and acid seems to evoke a synergistic effect to narrow the particle size distribution, while the subsequent addition of sucrose influences the dispersity index slightly (geraniol, citronellol) or not at all (*R*-limonene; see Figure 5J,K,L).

For recipe development, the aroma compounds geraniol, *R*-limonene, and citronellol were considered in order to achieve the greatest variance in droplet size obtained (geraniol: ∼190 nm; *R*-limonene: ∼180 nm; citronellol: ∼170 nm) for the subsequent investigations of fortification and translation into a continuous process.

##### 3.1.2.3 Oil Phase: Incorporation of Fish Oil

Incorporation of fish oil rich in omega-3 fatty acids (450 g/kg in the oil component) for fortification into the beverage emulsions’ composition (oil component: 100 g/kg aroma compound, 450 g/kg medium-chain triglycerides; aqueous phase: 100 g/L sucrose, citric acid with pH = 3) further eased surfactant diffusion during the emulsification, as demonstrated by overall smaller droplet sizes and dispersity indices (exception: geraniol). Compared to the nonfortified beverage emulsions, droplet size decreased by 33% from 191 to 127 nm (geraniol), by 15% from 174 to 147 nm (citronellol), and by 17% from 179 to 148 nm (*R*-limonene; see Figure 6A). In accordance, the droplet size distribution became narrower for fortified emulsions containing citronellol and *R*-limonene (dispersity index decreased by 8–15% to 0.23 for both), while the dispersity index of fortified emulsions containing geraniol was unaffected (see Figure 6B).

**Figure 6:**
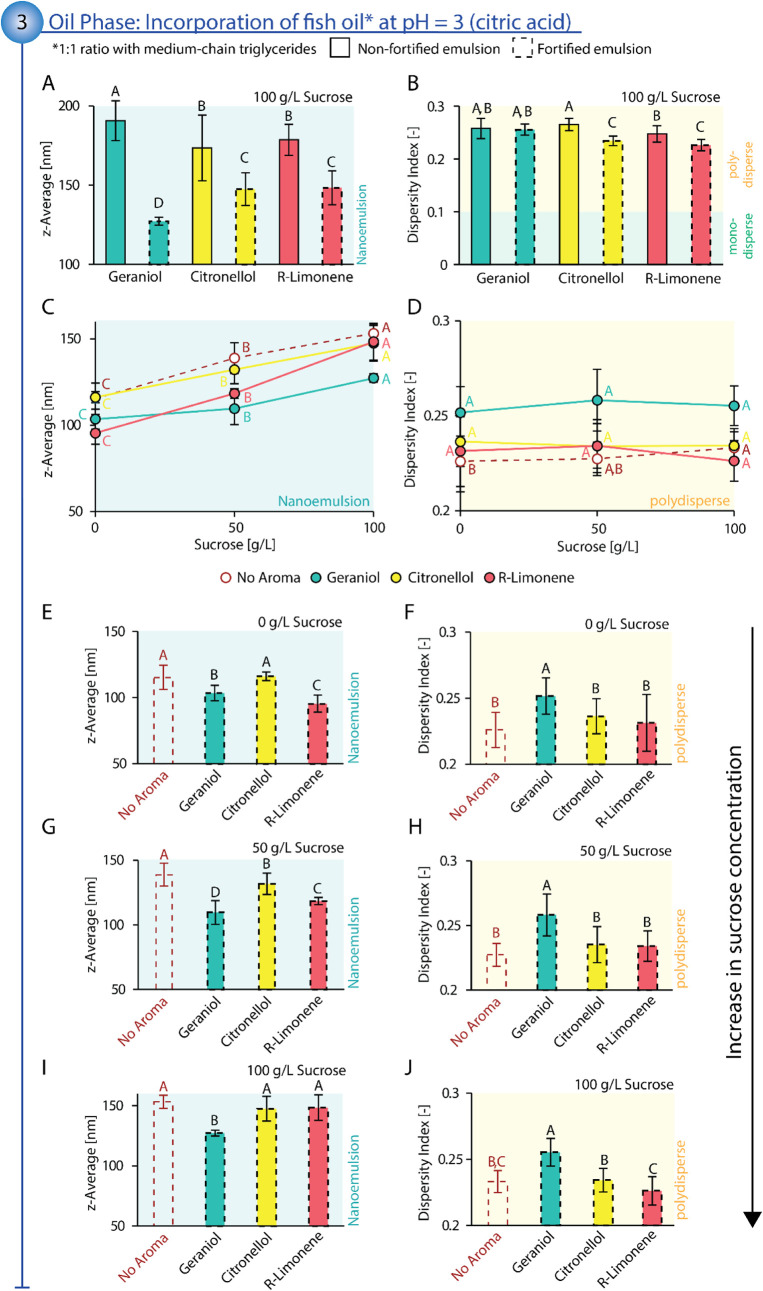
Top: Comparison of nonfortified, flavored emulsions (straight line) to fortified, flavored emulsions (dashed line) regarding average droplet size in the form of *z*-average (A) and dispersity index (B). Middle: Influence of sucrose concentration in the aqueous phase of fortified, nonflavored emulsions (dashed red line) and fortified, flavored emulsions (straight line) on average droplet size in the form of *z*-average (C) and dispersity index (D). Bottom: Comparison of fortified, nonflavored emulsions (red) to fortified, flavored emulsions at different sucrose concentrations regarding average droplet size in the form of *z*-average (0 g/L sucrose: (E), 50 g/L sucrose: (G), 100 g/L sucrose: (I)) and dispersity index (0 g/L sucrose: (F), 50 g/L sucrose: (H), 100 g/L sucrose: (J)). For nonfortified, flavored emulsions, the oil component consisted of medium-chain triglycerides (900 g/kg) and aroma compound (100 g/kg) mixed with the surfactant polysorbate 80 at an SOR of 1.0 to form the oil phase. For fortified, flavored emulsions, the oil component consisted of fish oil (450 g/kg), medium-chain triglycerides (450 g/kg), and aroma compound (100 g/kg) mixed with the surfactant polysorbate 80 at an SOR of 1.0 to form the oil phase. Aroma compounds used were geraniol (green), citronellol (yellow), and *R*-limonene (red) at a concentration to reach 100 ppm in the final emulsion. For all emulsions, the aqueous phase consisted of phosphate buffer (5 mM) and citric acid at pH = 3 at sucrose concentrations, as reported in the figures. All emulsions were prepared via the batch process at a 1 g/kg oil component content. Statistical significance was identified via ANOVA statistical testing (95% confidence interval) in combination with Tukey pairwise comparison. Statistically significant results are indicated by different letters, with “A” assigned to the overall highest value(s), “B” to the subsequent smaller value(s), and so forth. Reported are the average values with standard deviation.

Investigating the interplay of the three main ingredients previously identified to considerably affect emulsion characteristics (sucrose, aroma compound, and fish oil), sucrose remains a major driver in increasing droplet size (see Figure 6C). For each fortified emulsion (no aroma, geraniol, citronellol, *R*-limonene), droplet size increased consistently with increasing sucrose concentration. In contrast to previous results, the relative increase in droplet size from 0 g/L to 100 g/L sucrose was overall more pronounced than without fish oil (23–55% increase for fortified emulsions compared to 10–31% increase for nonfortified emulsions). While fish oil eases overall surfactant diffusion, the diffusion process is simultaneously more affected by the diffusion-hindering effect of sucrose. In alignment with previous observations of nonfortified emulsions, *R*-limonene seems to enhance the droplet size increase caused by sucrose (55% from 95 ± 6 nm at 0 g/L sucrose to 148 ± 11 nm at 100 g/L sucrose), while geraniol mitigates the relative increase (23% from 103 ± 6 nm at 0 g/L sucrose to 127 ± 2 nm at 100 g/L sucrose) compared to nonflavored fortified emulsions (33% from 115 ± 9 nm at 0 g/L sucrose to 153 ± 5 nm at 100 g/L sucrose). Similar to previous results, increasing sucrose concentration did not (geraniol, citronellol, and *R*-limonene) or only slightly (no aroma) affect the dispersity index of the fortified emulsions (see Figure 6D).

Comparing flavored to nonflavored fortified emulsions at different sucrose concentrations, only geraniol consistently led to an additional decrease in the droplet size (10–21% at 0–100 g/L sucrose; see Figure 6E,G,I). However, the addition of geraniol at the same time led to an increase in the dispersity index, generating a greater range of droplet sizes present in the emulsions (9–14% at 0–100 g/L sucrose; see Figure 6F,H,J). In contrast, the addition of citronellol led to almost identical emulsion characteristics compared to fish oil alone, while *R*-limonene only showed an additional decrease in droplet size at 0–50 g/L sucrose.

Although aroma compounds previously led to a general decrease in droplet size and dispersity indices compared to nonflavored, nonfortified emulsions, their effect can range from a further decrease in droplet size to a nonexisting effect for fortified emulsions, depending on the individual interaction with the fish oil. However, it needs to be noted that the observations might be influenced by the reduction of 50 g/kg of fish oil in the oil component required for the addition of 100 g/kg of aroma compound at a 1:1 ratio of fish oil to medium-chain triglycerides. Still, the observations demonstrate that careful selection of the oil component’s composition considerably influences the characteristics of the final emulsion. Only by changing the composition of the oil component, nanoemulsions instead of conventional emulsions were obtained at high sucrose concentrations. This effect should be systematically utilized to improve the emulsification efficiency of spontaneous emulsification. By identifying suitable aroma compounds and functional ingredients, the anticipated droplet size can be obtained while avoiding the need for specialized equipment or higher energy input, which are associated with higher costs and/or the risk of damaging sensitive bioactives. If no reduction in droplet size is required, the interactions of the oil components and emulsion characteristics can otherwise be utilized to decrease the surfactant concentration in the desired product.

### 3.2 Beverage Personalization via Recipe Library

We propose to achieve personalization of space beverages by establishing a recipe library to be implemented in an automated on-demand production line. The recipe library contains a wide range of beverage formulations or beverage recipes. These recipes differ in sweetness levels and flavor profiles to accommodate personal preferences in taste. Additionally, they differ in bioactive ingredients to cater to personal nutritional needs. When implemented in suitable software, astronauts (or, in the case of Earth application, the consumers) can choose their desired flavor, sweetness level, and bioactive ingredients from a range of options. The software can then access the associated beverage recipe to retrieve information on which “building blocks” (e.g., fortified, flavored oil phases and sweetened aqueous phases) are required to make the desired beverages. These building blocks can then automatically be fed into a continuous emulsification process, achieving on-demand, automated beverage production.

Using the results of the previous section, we designed six different fortified beverage recipes as a foundation for the beverage library (see Table 2). These beverage formulations differ in sweetness level (medium: 50 g/L, high: 100 g/L) and in aroma compounds (geraniol, citronellol, *R*-limonene). They all contain fish oil rich in omega-3 fatty acids as a bioactive ingredient, leading to a supplementation of ∼90 mg of omega-3 fatty acids per 330 mL beverage serving. The recipes can be achieved by using 5 building blocks: 2 building blocks of aqueous phase (medium and high sweetness) and 3 building blocks of oil phase (flavored with geraniol, citronellol, *R*-limonene). By incorporating more building blocks in the future, the range of beverage recipes can quickly be increased. For example, adding one building block to the aqueous phases (e.g., low sweetness) will lead to 9 recipes in total using 6 building blocks.

**Table 2:** Overview of the Six Different Recipes for Fortified Beverage Nanoemulsions as Determined by the Previous Sections

	Recipe 1	Recipe 2	Recipe 3	Recipe 4	Recipe 5	Recipe 6
Sweetener	Sucrose	Sucrose	Sucrose	Sucrose	Sucrose	Sucrose
Concentration [g/L]	50	100	50	100	50	100
Acid	Citric acid	Citric acid	Citric acid	Citric acid	Citric acid	Citric acid
pH	3	3	3	3	3	3
Aroma compound	Geraniol	Geraniol	Citronellol	Citronellol	*R*-Limonene	*R*-Limonene
Concentration^a^ [ppm]	100	100	100	100	100	100
Ratio of fish oil and MCT	1:1	1:1	1:1	1:1	1:1	1:1
Content oil component [g/kg]	1	1	1	1	1	1
SOR	1.0	1.0	1.0	1.0	1.0	1.0
*z*-Average [nm] (batch)	110 ± 9	127 ± 2	132 ± 8	147 ± 10	118 ± 3	148 ± 11
Dispersity index [-] (batch)	0.26 ± 0.02	0.26 ± 0.01	0.24 ± 0.01	0.23 ± 0.01	0.23 ± 0.01	0.23 ± 0.01

a Equal to a concentration of 100 g/kg in the oil component. MCT = medium-chain triglycerides. SOR = surfactant-to-oil ratio.

### 3.3 Continuous Beverage Production

#### 3.3.1 Preparation for Process Transfer to Continuous Microfluidic Process

##### 3.3.1.1 Ternary Phase Studies

A previous study in our lab on spontaneous emulsification involving emulsions made of medium-chain triglycerides, polysorbate 80, and phosphate buffer already demonstrated that at specific ratios between the three components, a gel would form.^32^ The presence of gel in a microfluidic process is critical, since, because of its semisolid properties, it can block the microchannels, interrupting the production and potentially causing equipment damage. Thus, critical phase behavior needs to be identified early on when transferring a process from batch to continuous mode. To investigate the phase behavior of the proposed fortified beverage recipes, ternary phase diagrams have been created for each recipe by varying the ratios between the 3 phases (aqueous phase, surfactant, and oil component) to identify critical states of the 3-phase systems. Compositions outside the final beverage emulsion are considered, as emulsions are often produced at a higher oil content to be subsequently diluted to application levels. In this case, low to medium contents of surfactant/oil (10–50%) and medium to high contents of the aqueous phase (40–80%) were investigated, as lower water contents will lead to water-in-oil emulsions instead of oil-in-water emulsions.

The six different 3-phase systems (see Figure 7A–F) behave almost identically regarding their phase behavior (appearance of emulsion and gel). Therefore, neither sucrose concentration nor the choice of aroma compounds influences the formation of emulsion or gel in these 3-phase systems. The driving compound in gel formation is the surfactant content. Once surfactant concentrations reach 30% and higher, gel formation begins. Depending on the composition, the gel observed during experimentation was either opaque or clear (see Figure 7G). Thus, gel formation begins at lower surfactant concentrations compared to our previous study, where gel formation only occurred at 30% surfactant concentrations in combination with low water content.^32^ For the determined recipes of this study, gel formation during the beverage production process can be avoided as long as the surfactant content is kept low (≤20 wt %) while water content is kept high (≥70 wt %).

**Figure 7:**
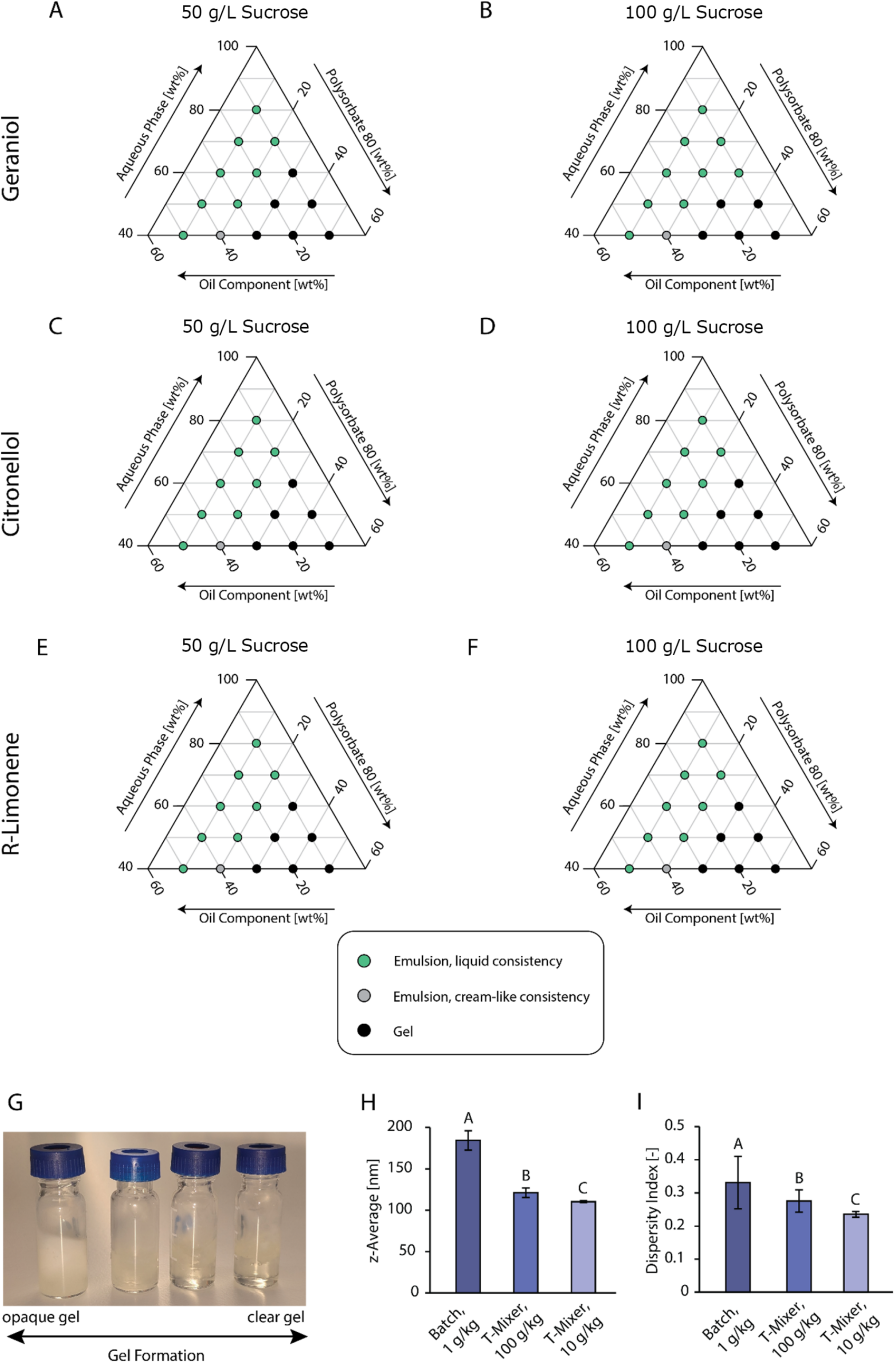
Top: Ternary phase studies of systems containing varied ratios of aqueous phase (5 mM phosphate buffer at a pH of 3, adjusted by citric acid with different sucrose concentrations), surfactant (polysorbate 80), and oil component (100 g/kg aroma compound, 450 g/kg medium-chain triglycerides, and 450 g/kg fish oil rich in omega-3 fatty acids). (A) System containing geraniol as the aroma compound at 50 g/L sucrose concentration in the aqueous phase. (B) System containing geraniol as the aroma compound at 100 g/L sucrose concentration in the aqueous phase. (C) System containing citronellol as the aroma compound at 50 g/L sucrose concentration in the aqueous phase. (D) System containing citronellol as the aroma compound at 100 g/L sucrose concentration in the aqueous phase. (E) System containing *R*-limonene as the aroma compound at 50 g/L sucrose concentration in the aqueous phase. (F) System containing *R*-limonene as the aroma compound at 100 g/L sucrose concentration in the aqueous phase. The oil component and surfactant were thoroughly mixed before adding the aqueous phase to ensure that emulsification via spontaneous emulsification could take place. Mixtures have been classified as gel or gel-like in consistency when viscosity was so high that intense shaking did not induce movement of the mixture. Bottom left: (G) Image depicting different occurrences of gel formation during ternary phase studies, ranging from opaque gel (left) to clear gel (right). Bottom right: Influence of process mode (batch, T-mixer) and oil content (g/kg) on average droplet size in the form of the *z*-average (H) and dispersity index (I) during the emulsification process; for the continuous microfluidic T-mixer, oil content refers to the oil content at the outlet of the process, which was diluted to 1 g/kg oil content before droplet size measurement. All emulsions contained 5 mM phosphate buffer at a pH of 7.4 as the aqueous phase and an oil phase of polysorbate 80 and food-grade medium-chain triglycerides at a SOR of 1.0. Statistical significance was identified via ANOVA statistical testing (95% confidence interval) in combination with Tukey pairwise comparison. Statistically significant results are indicated by different letters, with “A” assigned to the overall highest value(s), “B” to the subsequent smaller value(s), and so forth. Reported are the average values with standard deviation.

##### 3.3.1.2 Determination of Appropriate Volumetric Flow Rates and Process Parameters

In this study, the batch process was conducted by applying mass ratios between the different components. However, continuous processes are based on volume as a characteristic unit, since pumps displace a defined volume, thereby creating a volumetric flow rate in the connected channels. Thus, the density of the involved phases needs to be determined, which then allows the identification of appropriate volumetric flow rates, i.e., volumetric flow rates that generate the desired oil content in the final emulsion.

The density of an oil phase containing only food-grade medium-chain triglycerides and polysorbate 80 (SOR = 1.0) has been determined to be 1.002 ± 0.002 g/mL, while the addition of fish oil and an aroma compound to the oil component decreased the density of the resulting oil phase (SOR = 1.0) slightly by 1.4% in all cases (oil phase with geraniol: 0.990 ± 0.004 g/mL; oil phase with citronellol: 0.988 ± 0.006 g/mL; oil phase with *R*-limonene: 0.991 ± 0.004 g/mL). For the aqueous phase containing 5 mM phosphate buffer at a pH of 3, adjusted with citric acid, a density of 0.996 ± 0.001 g/mL was determined. Upon addition of sucrose, the density increased to 1.017 ± 0.001 g/mL at 50 g/L sucrose and to 1.035 ± 0.001 g/mL at 100 g/L sucrose.

In this study, the pumps available could not provide the accuracy required for a volumetric flow rate to obtain an emulsion with 1 g/kg oil content in the outlet. Therefore, emulsions were obtained at 100 or 10 g/kg oil content during continuous processing and diluted subsequently. The densities stated above allowed us to obtain the associated volume flows. Differences between the different oil phases and between the different aqueous phases turned out to be very small, i.e., in the range of 0.3–1.5%. Due to the low accuracy of the equipment, volumetric flow rates were kept constant at 2 and 8 mL/min (oil phase) for an emulsion containing 100 g/kg oil content and 0.2 and 9.8 mL/min (aqueous phase) for an emulsion containing 10 g/kg oil content.

Initial testing of the continuous microfluidic setup without beverage components demonstrated a considerate decrease in droplet size by 34–40%, depending on the oil content during emulsification (Figure 7H). In addition to the droplet size, the dispersity index also decreased considerably by 17–29% (see Figure 7I). Decreasing the oil content is likely to increase the relative surface area further, thus intensifying surfactant diffusion during spontaneous emulsification and leading to a smaller droplet size and a narrower droplet size distribution at 10 g/kg oil content compared to 100 g/kg oil content. A similar effect, i.e., a decrease in droplet size upon reduction in oil content, has been observed in batch.^69^ However, at 10 g/kg of oil content during emulsification, gel formation could be observed in the transparent channels transporting the oil phase to the T-mixer. Fortunately, the gel formation did not hinder emulsification but still increased the duration of the cleaning procedure (purging with ethanol). It is likely that at low volumetric flow rates, the aqueous phase can diffuse into the oil phase at the T-junction, forming the gel. In contrast, higher volumetric flow rates would lessen the diffusion of the aqueous phase into the oil phase at the T-junction, while also lessening the accumulation of gel as the affected oil phase would move more quickly through the channels. As this slight gel formation did not hinder the process, while leading to the overall smallest droplet size, the associated flow rates for 10 g/kg oil content in the outlet were still chosen to produce the beverages of the recipe library in the coming section. To reduce cleaning times in the future, it is advised to identify an optimum volumetric flow rate at which the smallest possible droplet size and the shortest possible cleaning time can be achieved.

#### 3.3.2 Process Transfer: Producing Fortified Beverage Nanoemulsions via Continuous Microfluidic Process

When comparing the droplet sizes of emulsions obtained via batch to emulsions obtained via the continuous microfluidic process (averaged over all six beverage recipes), the continuous process led to considerably smaller droplet sizes (a ∼28% decrease, see Figure 8A). The obtained nanoemulsions were in the range of 80–120 nm (depending on sucrose concentration) and hence had some of the smallest droplet sizes recorded in this study. The general decrease in droplet size confirms previous research^32^ as well as observations in the previous section (see Figure 7H). Changing the process mode, however, did not lead to an overall decrease in the dispersity index (see Figure 8B), as previously observed in Figure 7I. The dispersity index only decreased slightly at a sucrose concentration of 50 g/L but remained unaffected at a sucrose concentration of 100 g/L. A continuous microfluidic process can therefore still ease surfactant diffusion during spontaneous emulsification for more complexly formulated beverage products compared to simple model emulsions, while these findings suggest that the dispersity index is more influenced by emulsion composition than process mode.

**Figure 8:**
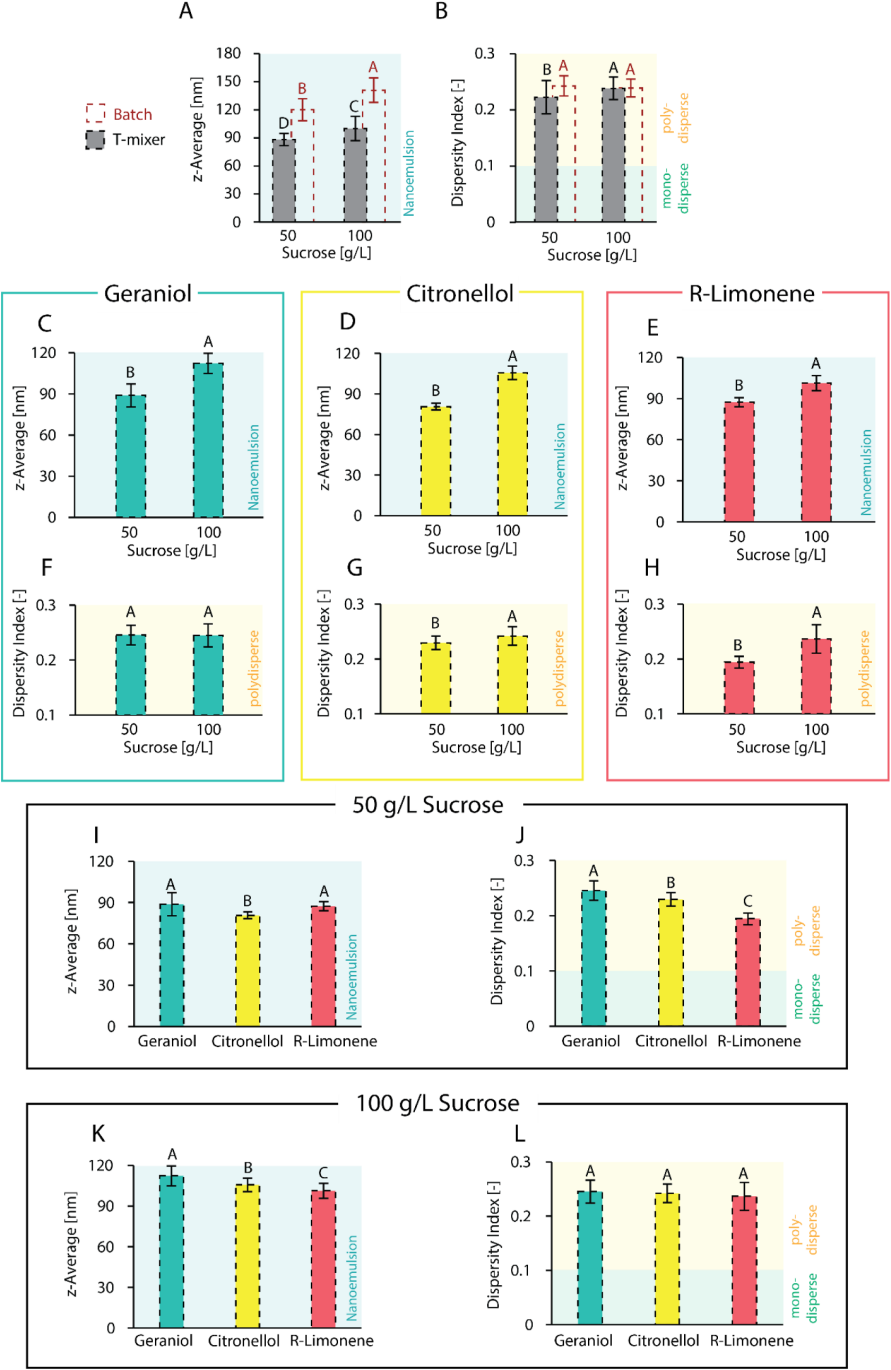
Top: Comparison of the batch process (red) to continuous microfluidic process (T-mixer, gray) as an emulsification process at different sucrose concentrations, focusing on average droplet size in the form of *z*-average (A) and dispersity index (B), both averaged over all six fortified, flavored beverage recipes. Middle: Influence of sucrose concentration on emulsion characteristics for fortified, flavored emulsions obtained via the T-mixer containing geraniol (*z*-average: C, dispersity index: F), citronellol (*z*-average: D, dispersity index: G), and *R*-limonene (*z*-average: E, dispersity index: H). Bottom: Comparison of different aroma compounds (geraniol, citronellol, *R*-limonene) in fortified, flavored emulsions obtained via the T-mixer at 50 g/L sucrose concentration (*z*-average: I, dispersity index: J) and 100 g/L sucrose concentration (*z*-average: K, dispersity index: L) in the aqueous phase. For fortified, flavored emulsions, the oil component consisted of medium-chain triglycerides (900 g/kg) and aroma compound (100 g/kg) mixed with the surfactant polysorbate 80 at an SOR of 1.0 to form the oil phase, and the aqueous phase consisted of phosphate buffer (5 mM) and citric acid at pH = 3, at sucrose concentrations as reported in the figures. For the batch process, emulsions were obtained at 1 g/kg oil component, and for the T-mixer process, emulsions were obtained at 10 g/kg and subsequently diluted to 1 g/kg oil content. Statistical significance was identified via ANOVA statistical testing (95% confidence interval) in combination with Tukey pairwise comparison. Statistically significant results are indicated by different letters, with “A” assigned to the overall highest value(s), “B” to the subsequent smaller value(s), and so forth. Reported are the average values with standard deviation.

Looking more closely at the emulsion composition, an increase in sucrose concentration led, in all cases, to an increase in droplet size (see Figure 8C–E), aligning with previous results. However, the relative increase in droplet size from 50 g/L sucrose to 100 g/L sucrose was often more pronounced (geraniol: increase of 26% vs 16% in batch; citronellol: increase of 16% vs 25% in batch; *R*-limonene: increase of 31% vs 12% in batch). While in batch, the increase in sucrose concentration mostly did not affect the dispersity index, for the continuous process, it mainly led to an increase in the dispersity index (22% increase for *R*-limonene, 5% increase for citronellol, no influence on geraniol; see Figure 8F,G,H). Even though the continuous process eases surfactant diffusion during spontaneous emulsification in general, there is a stronger dependence of droplet sizes and dispersity index on the sucrose concentration (Table 3).

**Table 3:** Overview of Emulsion Characteristics (Average Droplet Size in the Form of *z*-Average and Dispersity Index) of the Six Different Recipes for Fortified Beverage Nanoemulsions as Obtained via the Continuous Microfluidic Process (T-Mixer)

	Recipe 1	Recipe 2	Recipe 3	Recipe 4	Recipe 5	Recipe 6
Sucrose concentration [g/L]	50	100	50	100	50	100
Aroma compound	Geraniol	Geraniol	Citronellol	Citronellol	*R*-Limonene	*R*-Limonene
*z*-Average [nm] (T-mixer)	89 ± 8	112 ± 7	81 ± 2	106 ± 5	87 ± 3	101 ± 6
Dispersity index [-] (T-mixer)	0.25 ± 0.02	0.25 ± 0.02	0.23 ± 0.01	0.24 ± 0.02	0.19 ± 0.01	0.24 ± 0.03

Compared to the batch process, the individual aroma compounds demonstrated a different effect on the droplet size and dispersity index (see Figure 8I–L). While geraniol in the batch process led to the overall smallest droplet sizes at 50–100 g/L sucrose, for the continuous process, geraniol led to the overall largest droplet sizes and dispersity indices. Instead, the addition of citronellol or *R*-limonene caused a further decrease in droplet size (citronellol: 6–9% decrease at 50–100 g/L sucrose; *R*-limonene: 10% decrease at 100 g/L sucrose) and a decrease in dispersity at a sucrose concentration of 50 g/L. However, compared to the batch process, their relative influence on droplet size is smaller, resulting in an additional decrease in droplet size of only 6–11 nm. Thus, their influence on emulsion characteristics is likely to be negligible in contrast to process mode, fish oil content, and sucrose content.

In conclusion, both fortified beverage ingredients and the emulsification process can considerably influence spontaneous emulsification. For the investigated beverage ingredients, sucrose generally increased the droplet size with increasing concentration in the aqueous phase, while the addition of fish oil generally decreased the droplet size. Aroma compounds could influence emulsion characteristics under certain conditions, while acids had a negligible effect. Findings indicate that the ingredients’ changes in bulk phase characteristics either ease or hinder surfactant diffusion during spontaneous emulsification, while continuous emulsification increases the relative interfacial area, easing surfactant diffusion and decreasing droplet size. With process mode and fish oil being the key factors in increasing emulsification efficiency (amount of surfactant used in relation to droplet size), both showed an increase in sensitivity toward increasing sucrose concentration, as indicated by a higher relative increase in droplet size. Still, the results demonstrate that a strategic choice in beverage composition and process mode can design emulsion properties, even to the extent of transforming conventional emulsions into nanoemulsions. For common emulsification methods, the switch from macrodroplets to nanodroplets is typically achieved by increasing the input of mechanical energy. Applying spontaneous emulsification instead would save considerable amounts of mechanical energy. Our findings demonstrate that spontaneous emulsification can be a viable approach to producing fortified beverage nanoemulsions both in space and on the Earth.

Future investigations should involve in vivo and in vitro studies on food safety and sensory characteristics. While food-safe ingredients were used, cell and animal studies are the preferred option to rule out adverse health effects. Sensory studies on flavor release can ensure consumer acceptance. Especially, fish oil is known for its strong, fishy aroma, which might produce an unpleasant aftertaste when consumed in the emulsion form. Future stability studies should investigate whether the nanoemulsion formulation of omega-3 fatty acids provides sufficient protection against leakage or oxidation. The unsaturated double bonds react with air oxygen to form hydroperoxides, which are unstable and decompose into ketones, alcohols, and aldehydes.^70^ As thermodynamically unstable systems, emulsions will separate over time, and general emulsion stability investigations, i.e., monitoring particle size over long storage time, are advised. Preliminary stability investigations of the model emulsion (no addition of acid, sweetener, flavor, or bioactive) obtained via the continuous emulsification process showed no change in particle size after 2 days of storage at room temperature (see Figure 2 in the Supporting Information). Determination of the zeta potential can provide estimations of the droplets’ tendency to flocculate, although literature suggests that polysorbate 80 provides sufficient steric repulsion to overrule attractive van der Waals interactions.^20^ While microgravity is generally recognized to increase emulsion stability due to the absence of gravity-driven instability mechanisms such as creaming and sedimentation, high levels of radiation in space and high g-forces during launch can impact emulsion phase separation as well as general compound stability. As the opportunity to perform studies in space is limited, specialized facilities and equipment (radiation: on-ground particle accelerator facilities, high g-forces: centrifuge) can provide suitable analog conditions. The recipe library should be extended with a greater range of flavors and additional bioactives. Due to its lack of availability in standard astronaut nutrition, vitamin D is a suitable candidate for beverage fortification. Furthermore, our findings show the potential for a future reduction in surfactant concentration, which itself does not provide any nutritional benefit. Due to the strong influence of surfactant concentration on droplet size,^30,32^ reducing surfactant concentration comes with a trade-off of larger droplet sizes, which could be mitigated by the increased emulsification efficiency via conscious emulsion formulation and/or process mode. Especially, the positive results of the continuous microfluidic process are promising for a further increase in emulsification efficiency following process optimization. Future process optimization comprises in-depth evaluation of volumetric flow rates as a measure to minimize issues with jellification, as well as exploring more advanced internal mixer geometries. Compared to the simple T-mixer, microfluidic mixers with changing diameters (e.g., by narrowing and widening of channels, small mixing chambers) and advanced flow patterns (e.g., zigzag pathway, coiled flow inverter) can lead to a greater relative interfacial area and higher shear rates at the point where the oil and aqueous phases initially mix and increase mixing diagonally to the liquid flow. These measures are likely to increase emulsification efficiency by supporting the diffusion of the surfactant, while potentially decreasing overall process time by reducing postemulsification stirring times.

## Supplementary Material



## Notes

Comparison of different qualities of medium-chain triglycerides regarding their impact on spontaneous emulsification, stability evaluation of the model emulsion after 2 days, particle size distributions of the emulsions, and supportive information for the vial preparation for the ternary phase studies (4 figures and 2 tables, 5 pages in total) (PDF)
