# Supplementary material for: Formulation of Personalized, Fortified Beverage Nanoemulsions for Space Exploration with Omega‑3 Polyunsaturated Fatty Acids
Source: ACS Food Sci Technol. 2026 Apr 8;6(4):1092–111. doi: 10.1021/acsfoodscitech.5c01291 (PMC13097132; doi:10.1021/acsfoodscitech.5c01291)
Supplement: Supplementary file 1 [file fs5c01291_si_001.pdf]

Supplementary Materials

For

Formulation of personalized, fortified beverage nanoemulsions for space exploration with omega-3 polyunsaturated fatty acids

Svenja Schmidt[1,2,3,4]*, Ian D. Fisk[2,4,5], Nicole Yang[4], Maria Saarela[6,7], Volker Hessel[1,2,3]*

[1] School of Chemical Engineering, Adelaide University, Adelaide, 5005, Australia.

[2] ARC Centre of Excellence in Plants for Space, Adelaide University, Waite Campus, Urrbrae, 5064, Australia.

[3] Andy Thomas Centre for Space Resources, Adelaide University, Adelaide, 5005, Australia.

[4] International Flavour Research Centre, University of Nottingham, Sutton Bonington Campus, Loughborough, LE12 5RD, United Kingdom.

[5] International Flavour Research Centre (Adelaide), Adelaide University, Waite Campus, Urrbrae, 5064, Australia.

[6] South Australian Research and Development Institute (SARDI), Adelaide, 5001, Australia.

[7] School of Agriculture, Food, and Wine, Adelaide University, Waite Campus, Urrbrae, 5064, Australia.

* Email: volker.hessel@adelaide.edu.au; svenja.schmidt@adelaide.edu.au; svenja.schmidt@nottingham.ac.uk

Summary of Contents

The Supplementary Materials for the presented study contains 4 figures and 2 tables (5 pages total). Additional information is given on the quality of used carrier oil (Figure 1), stability of the emulsions (Figure 2), the particle size distributions of the emulsions (Figure 3), and on the vial preparation within the ternary phase studies (Figure 4, Table 1 and 2).

Table of Contents

1. Supplementary materials regarding the emulsification 1
2. Supplementary materials regarding emulsion stability 1
3. Supplementary materials regarding emulsion characteristics 2
4. Supplementary materials regarding the ternary phase studies 4

1. Supplementary materials regarding the emulsification

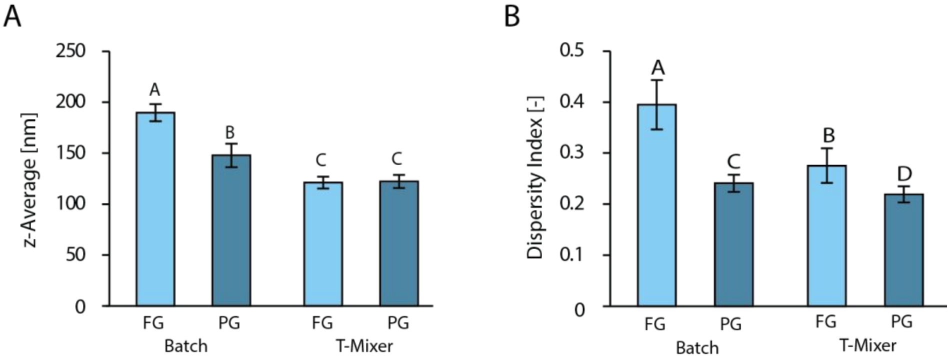

Figure 1: Comparison between food-grade (FG) and pharmaceutical grade (PG) medium-chain triglycerides on nanoemulsion characteristics (A: z-Average and B: dispersity index) formed via spontaneous emulsification in batch process and continuous, microfluidic process (T-Mixer). All emulsions contained 5 mM phosphate buffer and polysorbate 80 as surfactant in the oil phase at a SOR of 1.0. For the T-mixer, emulsions were obtained at 100 g/kg oil content and subsequently diluted to 1 g/kg oil content, while in batch, emulsions were directly obtained at 1 g/kg oil content. Statistical significance was identified via ANOVA statistical testing (95% confidence interval) in combination with Tukey pairwise comparison. Statistically significant results are indicated by different letters, with 'A' assigned to the overall highest value(s), 'B' to the subsequent smaller value(s) and so forth. Reported are the average values with standard deviation.

2. Supplementary materials regarding emulsion stability

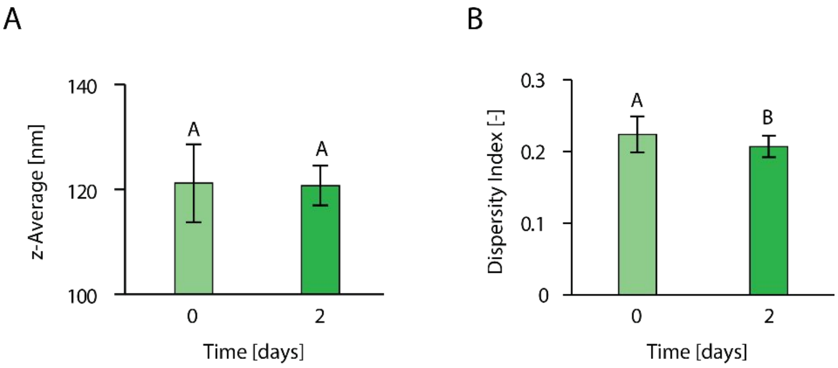

Figure 2: Influence of short-term storage (2 days) on z-Average (A) and dispersity index (B). Daz 0 describes the time directly after the emulsification process and prior to storage in an upright position at room temperature. All emulsions contained 5 mM phosphate buffer as aqueous phase and polysorbate 80 and pharmaceutical grade medium-chain triglycerides as oil phase at a SOR of 1.0. All emulsions were obtained via a continuous microfluidic process (T-mixer) at 100 g/kg oil content and were subsequently diluted to 1 g/kg oil content. Emulsion preparation and storage was prepared 10 times. Statistical significance was identified via ANOVA statistical testing (95% confidence interval) in combination with Tukey pairwise comparison. Statistically significant results are indicated by different letters, with 'A' assigned to the overall highest value(s), 'B' to the subsequent smaller value(s) and so forth. Reported are the average values with standard deviation.

3. Supplementary materials regarding emulsion characteristics

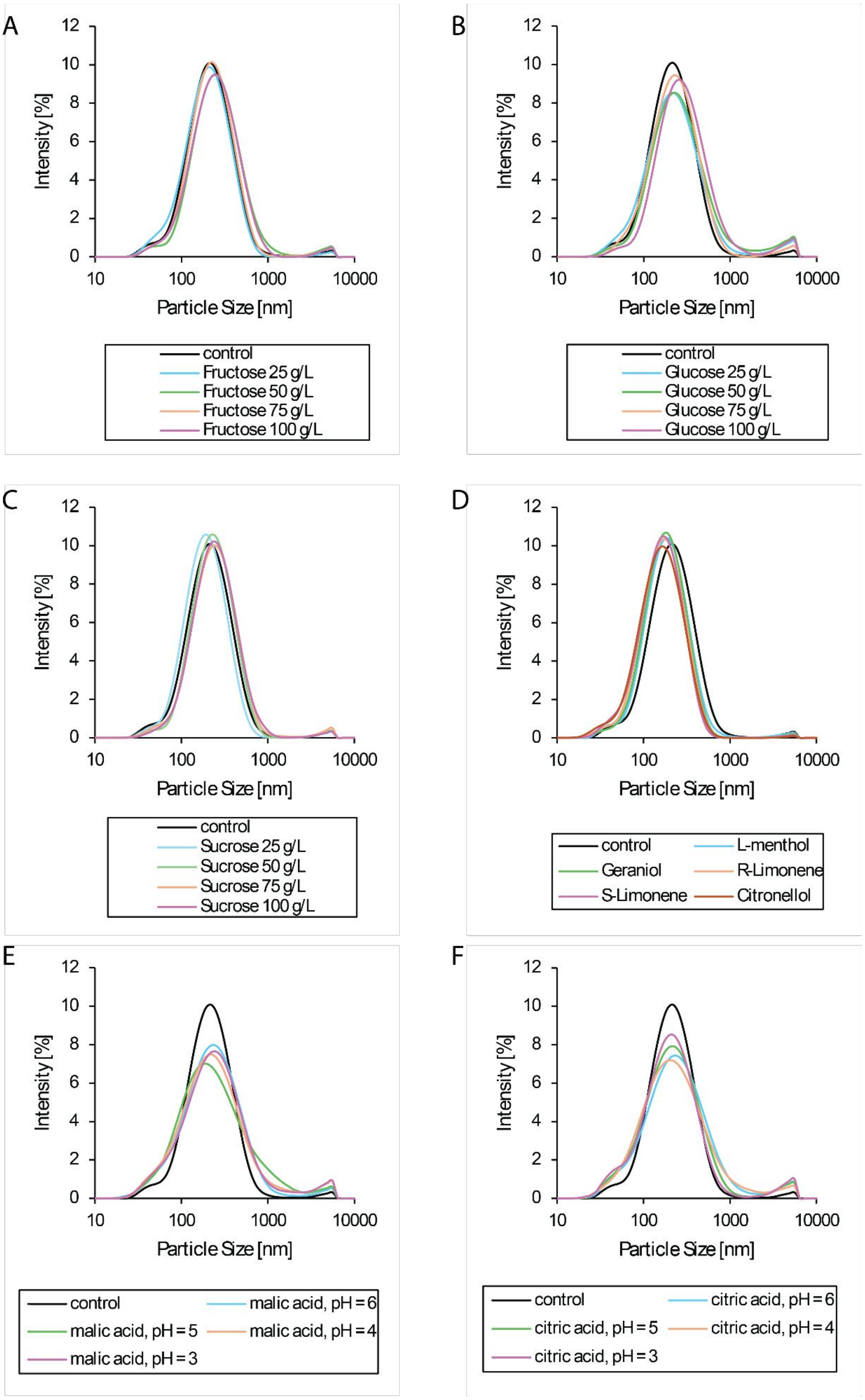

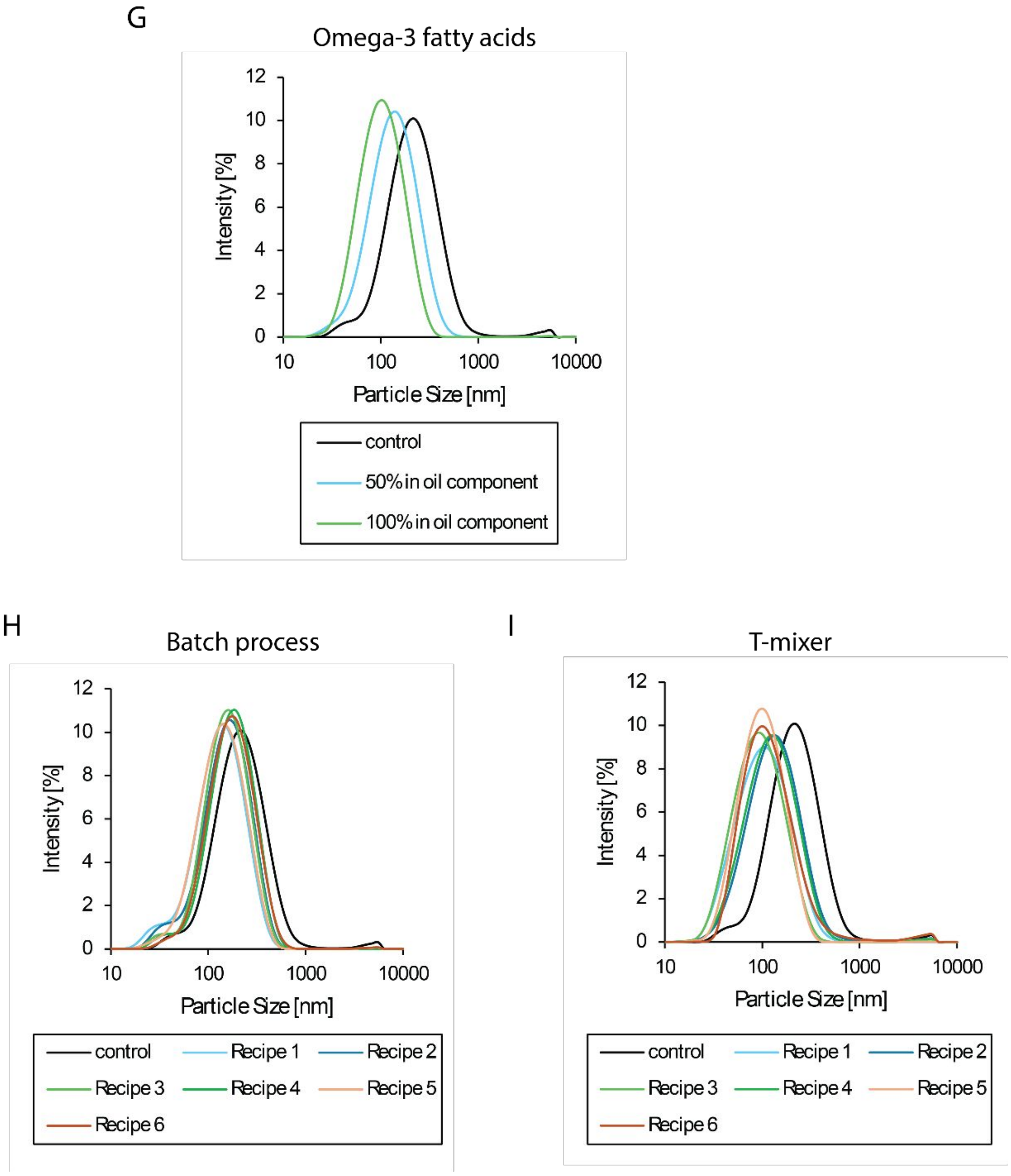

Figure 3: Influence of fructose concentration (A), glucose concentration (B), sucrose concentration (C), type of aroma compound (D), pH adjusted with malic acid (E), pH adjusted with citric acid (F), and concentration of omega-3 fatty acids (G) on the particle size distributions; (A-C, E, F) describe beverage ingredients in the aqueous phase and (D, G) describe beverage ingredients in the oil phase; Influence of batch process (H) and T-mixer (I) on the particle size distribution of the 6 identified recipes for personalised fortified beverage library; for (A-I), control relates to the model emulsion (5 mM phosphate buffer at pH = 7.4, polysorbate 80, medium-chain triglycerides) obtained via the batch process. All emulsions contained 5 mM phosphate buffer as aqueous phase and polysorbate 80 mixed with medium-chain triglycerides as oil phase. Additional beverage ingredients were added as indicated and the composition of the recipes is recorded in Table 2 in the main text. For the T-mixer, emulsions were obtained at 100 g/kg oil content and subsequently diluted to 1 g/kg oil content, while in batch, emulsions were directly obtained at 1 g/kg oil content. Reported are the average values of the particle size distributions.

4. Supplementary materials regarding the ternary phase studies

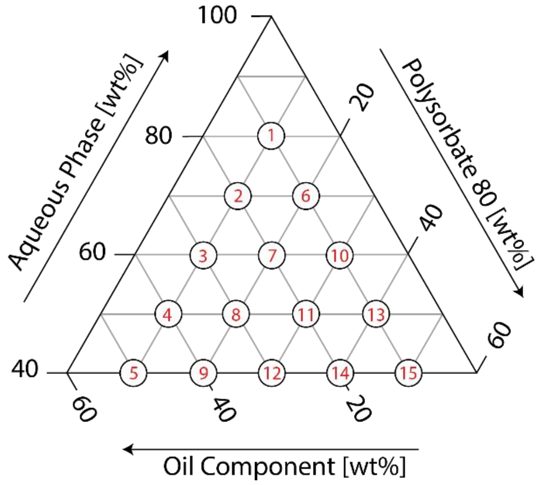

Figure 3: Schematic showing the area investigated for the different 3-phase systems (aqueous phase, oil component = mixture of medium-chain triglycerides, omega-3 fatty acids, and aroma compound, polysorbate 80 as surfactant), numbers indicate the number of the vial in Table 1.

Table 1: Overview of vials prepared for the ternary phase system studies showing the composition of each vial; m is the mass of the respective constituent.

# Vial	$m_{oil\ component}$ [g]	$m_{surfactant}$ [g]	$m_{aqueous\ phase}$ [g]
1	0.1	0.1	0.8
2	0.2	0.1	0.7
3	0.3	0.1	0.6
4	0.4	0.1	0.5
5	0.5	0.1	0.4
6	0.1	0.2	0.7
7	0.2	0.2	0.6
8	0.3	0.2	0.5
9	0.4	0.2	0.4
10	0.1	0.3	0.6
11	0.2	0.3	0.5

12	0.3	0.3	0.4
13	0.1	0.4	0.5
14	0.2	0.4	0.4
15	0.1	0.5	0.4

Table 2: Overview of the composition of the different 3-phase systems studied for the ternary phase system studies; for simplicity, the composition of the oil component is shown for a mass of 10 g and m is the mass of the respective ingredient.

	3-Phase System 1	3-Phase System 2	3-Phase System 3	3-Phase System 4	3-Phase System 5	3-Phase System 6
Composition of 10 g of the Oil Component, $m_{Ingredient}$ [g]						
MCT	4.5	4.5	4.5	4.5	4.5	4.5
O3FA	4.5	4.5	4.5	4.5	4.5	4.5
Geraniol	1	1	-	-	-	-
Citronellol	-	-	1	1	-	-
R-Limonene	-	-	-	-	1	1
Composition of the Aqueous phase						
Sucrose [g/L]	50	100	50	100	50	100
Surfactant						
Polysorbate 80	✓	✓	✓	✓	✓	✓
